# Assessing the prognostic and therapeutic value of cuproptosis-related genes in colon adenocarcinoma patients

**DOI:** 10.3389/fcell.2025.1550982

**Published:** 2025-04-10

**Authors:** Zhanhui Ye, Yixian Song, Mengqing Zhu, Fuying Zheng, Wenjie Qin, Xue Li, Pei Wang, Zihua Li, Kequan Chen, Aimin Li

**Affiliations:** ^1^ Guangdong Provincial Key Laboratory of Gastroenterology, Department of Gastroenterology, Nanfang Hospital, Southern Medical University, Guangzhou, China; ^2^ Endoscopy Center, Jiangmen Central Hospital, Jiangmen, China; ^3^ Department of Anesthesia, Critical Care and Pain Medicine, Massachusetts General Hospital, Harvard Medical School, Boston, MA, United States; ^4^ Department of Orthopedics, Shanghai Tongji Hospital, School of Medicine, Tongji University, Shanghai, China; ^5^ Department of Gastroenterology, The First Affiliated Hospital of Guangzhou Medical University, Guangzhou, China

**Keywords:** colon adenocarcinoma, cuproptosis-related genes, prognostic risk model, tumor microenvironment, ferredoxin 1, elesclomol, cuproptosis

## Abstract

**Background:**

Colon adenocarcinoma (COAD) remains a major global health challenge with poor prognosis despite advances in treatment, underscoring the need for new biomarkers. As a novel mode of cell death, cuproptosis is thought to be potentially involved in the development of cancer. However, the particularly as the role of cuproptosis-related genes (CRGs) in COAD prognosis and therapy remains unclear.

**Methods:**

We analyzed RNA sequencing data from The Cancer Genome Atlas for COAD, focusing on CRG expression patterns and their clinicopathological correlations. Using the Weighted Gene Co-expression Network Analysis (WGCNA) method, we identified the gene module most strongly linked to cuproptosis and conducted functional enrichment analysis to explore the roles of genes within this module in COAD tumorigenesis. A novel prognostic risk model based on four CRGs (ORC1, PTTG1, DLAT, PDHB) was developed to stratify COAD patients into high-risk and low-risk groups, assessing overall survival, tumor microenvironment, and mutational landscape differences. We also evaluated the therapeutic effects of ferredoxin 1 (FDX1) and elesclomol in promoting cuproptosis in HCT116 and LoVo cell lines through various experiments, including cell proliferation, apoptosis assessment, mitochondrial membrane potential evaluation, and DLAT lipoylation detection via Western blot.

**Results:**

Certain CRGs showed different expressions in COAD *versus* normal tissues. WGCNA identified a gene module linked to cuproptosis, crucial for pathways like cell cycle regulation, citrate cycle (TCA cycle), and DNA replication. The novel risk model stratified patients into high and low-risk groups based on risk scores, revealing that high-risk COAD patients had shorter overall survival and distinct immune cell infiltration, while low-risk patients were more sensitive to immunotherapy. Experimental results indicated that FDX1 exerted an inhibitory effect on COAD, and its combination with elesclomol significantly reduced proliferation, promoted apoptosis, increased DLAT lipoylation, and lowered mitochondrial membrane potential in COAD cells.

**Conclusion:**

The findings of this study provided a new perspective for the research on biomarkers and therapeutic strategies in COAD, evaluated the prognostic and therapeutic value of CRGs in COAD patients, and laid a theoretical foundation for the future clinical application of CRGs.

## 1 Introduction

Colon adenocarcinoma (COAD) ranks among the most prevalent and lethal cancers worldwide, imposing significant psychological and financial burdens on patients and their families (Bray et al., 2024). Numerous factors contribute to COAD development, including family history, genetic mutations, prior disease history, obesity, and dietary habits (Khan et al., 2022). Despite advancements in early diagnosis, surgical techniques, and chemoradiotherapy over the past several decades, the prognosis for COAD patients remains unsatisfactory due to high recurrence and mortality rates (Collaborators, 2022). Therefore, identifying new biomarkers and therapeutic targets for COAD is of paramount importance.

A 2022 *Science* study discovered a novel cell death mechanism termed cuproptosis, wherein intracellular copper accumulation causes abnormal aggregation of lipoylated proteins in the tricarboxylic acid (TCA) cycle, disrupts Fe-S cluster proteins involved in mitochondrial respiration, triggers a protein toxicity stress response, and ultimately leads to cell death that cannot be rescued by inhibitors of other known cell death pathways such as apoptosis, necroptosis, oxidative stress-induced cell death, or ferroptosis (Tsvetkov et al., 2022). Cuproptosis has been closely associated with the development of many cancers. For instance, in gastric cancer, it promotes lactylation at the K229 site of METTL16, which in turn mediates the m^6^A modification on FDX1 mRNA, thereby enhancing cuproptosis (Sun et al., 2023). Additionally, the ferroptosis inducers sorafenib and erastin promote cuproptosis in primary hepatocellular carcinoma by inhibiting FDX1 protein degradation via inhibition of cystine transport and by decreasing synthesis of the intracellular copper chelator GSH (Wang et al., 2023). These findings underscore the importance of cuproptosis induction as a promising cancer therapeutic strategy. However, few studies have addressed the specific molecular mechanisms of cuproptosis in COAD, leaving its role in this context unclear.

It has been proved that across diverse cancer cell lines, the occurrence of cuproptosis relies on Cu^+^ causing the oligomerization of lipoylated dihydrolipoamide S-acetyltransferase (DLAT) in the mitochondria, while the reduction of Cu^2+^ into the more toxic Cu^+^ and the lipoylation of DLAT depend on FDX1 (Tsvetkov et al., 2022; 2019). Consequently, FDX1 plays a vital role in the mechanisms underlying cuproptosis. A previous study has shown that FDX1 expression is significantly reduced in COAD tissues compared to normal tissues, and COAD patients with high FDX1 expression have better overall survival compared to those with low FDX1 expression (L. Wang et al., 2023). Another study suggests that FDX1 inhibits the growth and progression of COAD by suppressing epithelial-mesenchymal transition (EMT) (Wang et al., 2025). Except for FDX1, copper ionophore elesclomol emerges as another crucial factor in inducing cuproptosis, as reported in the study published in *Science* (Tsvetkov et al., 2022). Elesclomol has been confirmed to enhance the inhibitory effect on COAD cells when combined with 4-octyl itaconate (4-OI), which promotes the inhibition of aerobic glycolysis by targeting glyceraldehyde 3-phosphate dehydrogenase (GAPDH) (Yang et al., 2023). After treatment with elesclomol-Cu^2+^; E2F3 expression in COAD cells increases, significantly enhancing their resistance to elesclomol (Zhou et al., 2023). Nonetheless, research on the combined effect of FDX1 and elesclomol in enhancing cuproptosis in COAD is still lacking.

To assess the prognostic value of cuproptosis-related genes (CRGs) and understand how cuproptosis influences COAD, we conducted an in-depth analysis of COAD data from The Cancer Genome Atlas (TCGA). Initially, we identified the module most relevant to cuproptosis through Weighted Gene Co-expression Network Analysis (WGCNA) and investigated the roles of these genes in the tumorigenesis of COAD through functional analysis. Subsequently, identified CRGs significantly impacting patient prognosis and constructed a novel prognostic risk model based on these genes. By stratifying COAD patients into high-risk and low-risk groups according to the model, we performed functional analysis, immune infiltration evaluation, and mutational profiling on both groups to unveil the roles of CRGs in COAD development and immune regulation. Lastly, to address the gap in research on the combined effects of FDX1 and elesclomol in enhancing cuproptosis levels in COAD, we investigated their joint impact on COAD cell proliferation, apoptosis, and cuproptosis levels. By focusing on the key cuproptosis gene FDX1, we elucidated its role in cuproptosis and assessed its therapeutic potential in COAD. Collectively, our study offers promising new strategies for assessing the prognostic and therapeutic value of CRGs in patients with COAD.

## 2 Materials and methods

### 2.1 Regents

Cell culture medium RPMI-1640 (PM150110) and Ham’s F-12K (PM150910) were purchased from Procell. Fetal Bovine Serum (FSP500) was obtained from ExCell Biological Technology. Antibody against Lipoic Acid (ab58724) was sourced from Abcam, while antibodies against FDX1 (12592-1-AP) and β-actin (66009-1-Ig) were purchased from Proteintech. Polyvinylidene fluoride (PVDF) membranes (IPVH00010) were supplied from Millipore. Elesclomol (STA-4783) was acquired from MCE. Copper(II) chloride anhydrous (C804817) was purchased from Macklin. Transfection Regent Lipofectamine 3000 (L3000015) was obtained from Thermo Scientific. RNA Extraction Reagent (AG21101) and *Evo M-MLV* Reverse-transcription-kit (AG11706) were sourced from Accurate Biology. SYBR Green qPCR Mix (RK21203) was procured from ABclonal. From Beyotime, the following were purchased: Penicillin-Streptomycin Solution (C0222), RIPA Lysis Buffer (P0013C), ECL reagent (P0018M), Cell Counting Kit-8 (CCK-8) solution (C0039), Annexin V-FITC Apoptosis Detection Kit (C1062L), 4% Paraformaldehyde Fix Solution (P0099), Crystal Violet Staining Solution (C0121), five-ethynyl-2' -deoxyuridine (EdU) Cell Proliferation Kit with Alexa Fluor 488 (C0071L), and Tetramethylrhodamine, ethyl ester (TMRE, C2001S).

### 2.2 Retrieval of TCGA-COAD RNA-Seq and clinical data

Data from The Cancer Genome Atlas (TCGA), including RNA-sequencing data (Fragments Per Kilobase of transcript per Million mapped reads, FPKM values) and matched clinical information of COAD in TCGA-COAD cohort, were retrieved from the University of California Santa Cruz (UCSC) Xena database (https://xenabrowser.net/datapages/?cohort=GDC%20TCGA%20Colon%20Cancer%20(COAD)&removeHub=https%3A%2F%2Fxena.treehouse.gi.ucsc.edu%3A443).

### 2.3 Expression and WGCNA-based mechanistic insights of CRGs

Based on the research published in *Science* (Tsvetkov et al., 2022), CRGs include FDX1, LIPT1, LIAS, DLD, DBT, GCSH, DLST, DLAT, PDHA1, PDHB, SLC31A1, ATP7A, and ATP7B. Using the R packages ‘clusterProfiler’ and ‘limma’, we extracted the expression levels of CRGs from the TCGA-COAD dataset. Box plots were generated using the ggboxplot function to compare the expression levels of CRGs in normal tissues *versus* COAD tissues. The Wilcoxon test was employed to assess the differences between the two groups. Next, COAD sample information lacking detailed clinical staging data, specifically the Tumor (T) stage, Node (N) stage, and Metastasis (M) stage, was removed. Subsequently, the Analysis of Variance (ANOVA) method was employed to statistically compare the expression levels of CRGs across these different stages.

Using the WGCNA (version 1.70) R package, we processed and conducted an in-depth analysis of the TCGA-COAD dataset. The Gene Set Variation Analysis (GSVA, version 1.22.4) algorithm was employed to calculate the activity scores of the dataset, which were referred to as GSVA scores. Sample clustering was conducted, a sample tree was plotted and trimmed, and the plotDendroAndColors function was utilized to integrate the sample clustering dendrogram with the heatmap of GSVA scores. Calculated the number of the module eigengenes and samples, recomputed the module eigengenes using color labels, and determined the correlation between these eigengenes and cuproptosis. After identifying the module with the strongest correlation with cuproptosis, the top 100 genes with the highest connectivity from that module were selected. To further explore the potential role of 13 CRGs and the top 100 genes within the magenta modules in the tumorigenesis of COAD, we conducted functional enrichment analyses. Specifically, we performed Gene Ontology (GO) and Kyoto Encyclopedia of Genes and Genomes (KEGG) analyses to identify the biological functions of these genes. The GO categories encompassed three terms: cellular component (CC), biological process (BP), and molecular function (MF). For all enrichment analyses, we utilized the ‘clusterProfiler’ and ‘limma’ packages in R software, based on background genes derived from the GO and KEGG databases (Ritchie et al., 2015; Yu et al., 2012).

### 2.4 Construction of a prognostic risk model based on CRGs

Based on the top 100 genes with the highest connectivity in the cuproptosis-related module identified through WCGNA and 13 CRGs, univariate Cox regression analysis was conducted to screen for genes related to prognosis. Subsequently, the selected genes underwent least absolute shrinkage and selection operator (LASSO) Cox regression analysis, and genes with relatively larger weights were identified for the construction of a prognostic risk model for COAD patients (Fu and Wang, 2018; Ma et al., 2022; Strobl et al., 2008). Using the model, we calculated the risk score for each COAD patient and then classified them into high-risk and low-risk groups based on the median risk score. We then validated the prognostic significance of the risk score signature through univariate and multivariate Cox proportional hazards regression analyses, incorporating clinical parameters. Based on the two groups characterized by the risk score, we identified the differentially expressed genes (DEGs) between them. These DEGs were visualized in the form of volcano plots using the ‘ggplot’ R package. With a fold-change threshold set at 1 and an adjusted *p*-value threshold at 0.05, we employed the ‘clusterProfiler’ R package to perform enrichment analyses, including GO and KEGG analyses, to explore potential correlated enrichment terms. Additionally, Gene Set Enrichment Analysis (GSEA) was conducted to analyze changes in pathway levels between high-risk and low-risk groups.

### 2.5 Analysis of the relationship between tumor microenvironment and risk scores

To analyze the relationship between tumor microenvironment and risk scores, we first applied CIBERSORT (Cell type Identification By Estimating Relative Subsets Of RNA Transcripts), a deconvolution algorithm, and single-sample GSEA (ssGSEA) analyses to quantify the levels of tumor immune infiltration and determine the proportion of immune cells in each sample(Newman et al., 2015). The immune cell abundance between high-risk and low-risk groups was analysed using Wilcoxon test. Within the high-risk and low-risk groups, we further examined the expression differences of representative immune checkpoint molecules using a Wilcoxon rank-sum test. The cytolytic activity (CYT) score for each sample in both the high-risk and low-risk groups was obtained by calculating the geometric mean of GZMA and PRF1 gene expression. Furthermore, the tumor immune estimation resource (TIMER) (http://timer.cistrome.org), which molecular characterization of 32 types of cancer and six types of immune cells, was used to investigate the tumor microenvironment and the correlation between four hub CRGs in the risk model and various immune cell types(Bindea et al., 2013; Li et al., 2017) Finally, to assess the relationship between tumor purity and risk scores, we employed the ESTIMATE (Estimation of STromal and Immune cells in MAlignant Tumours using Expression data) algorithm to estimate the abundance of stromal and immune cells in malignant tumors (Bindea et al., 2013).

### 2.6 Mutation landscape analysis

The landscape of top mutated genes between the high-risk and low-risk groups was presented with mutation types and frequencies, and data analysis was performed by the ‘maftools’ package (Version 2.12.0).

### 2.7 Clinical colon adenocarcinoma samples

The Ethics Committee of Nanfang Hospital approved the collection of cancerous and adjacent non-cancerous tissues for this study. All specimens were confirmed to be COAD through pathological validation. Additionally, none of the patients received preoperative neoadjuvant radiotherapy, nor did they have multiple tumors. Fresh surgical specimens were either quickly frozen in liquid nitrogen and stored at −80°C or embedded in paraffin to create wax blocks.

### 2.8 Cell culture and treatment

Human COAD cell lines including HCT116 and LoVo cells were obtained from American Type Culture Collection (ATCC). HCT116 cells were cultured in a medium containing 89% RPMI-1640 medium, 10% Fetal Bovine Serum (FBS), and 1% Penicillin-Streptomycin (P-S). LoVo cells were cultured in a medium containing 89% Ham’s F-12K medium, 10% FBS, and 1% P-S. Kidan Biosciences (Guangzhou, China) designed and synthesized the FDX1 plasmid. Plasmid transfection was performed using Lipofectamine 3000 Regent according to the instructions. To induce cuproptosis, COAD cells were pulse-treated for 2 h with a combination of 100 nM elesclomol (ES) and 2 μM CuCl_2_. To inhibit cuproptosis induced by ES, COAD cells were treated with a 2-h pulse of 100 nM ES and 2 μM CuCl_2_ in combination with 20 μM of the cuproptosis inhibitor tetrathiomolybdate (TTM). To Cell morphology was observed under light microscopy after treatment.

### 2.9 Real-time quantitative PCR analysis

Total RNA was isolated and extracted from either tissues or cells utilizing the RNA Extraction Reagent. A NanoDrop LITE spectrophotometer (Thermo Scientific) was used to assess the concentration and integrity of the RNA. Reverse transcription was performed with the *Evo M-MLV* Reverse-transcription kit according to the manufacturer’s instructions. The resultant complementary DNA (cDNA) was analyzed through real-time quantitative PCR (RT-qPCR). Data analysis was conducted using the ^ΔΔ^CT methodology for comparative analysis of relative mRNA expression. ACTB (beta actin) was used as the internal reference. The primers utilized for RT-qPCR amplification are detailed in Supplementary Table S1.

### 2.10 Cell proliferation analysis

Cell proliferation was detected using clone formation assays, CCK-8 assays and EdU proliferation assays. The clone formation assays started with seeding cells in 12-well plates and culturing them until colonies formed after treatment. Then, the cells were fixed using 4% paraformaldehyde, stained with crystal violet, photographed, and counted. The experimental method for CCK-8 assays involved adding 10 μL of CCK-8 solution to each well of the treated 96-well plate containing cells. The plate was then incubated in the dark for 2 h, followed by measuring the absorbance at 450 nm to indicate the cell count through a linear relationship. EdU proliferation assays were conducted using the EdU Cell Proliferation Kit with Alexa Fluor 488. The samples were stained and then observed under a fluorescence microscope (OLYMPUS BX53), revealing that proliferating cells exhibited bright green fluorescence.

### 2.11 Apoptosis analysis

After digesting the treated cells, 1 × 10^5^ cells from each treatment group were collected and subsequently stained with 5 µL of Annexin V and 10 µL of propidium iodide (PI). Following a 20-min incubation in the dark at room temperature, the stained cells were analyzed using flow cytometry (Beckman Coulter).

### 2.12 Mitochondrial membrane potential measurement

After treatment, live cells were stained with 10 μM TMRE for 30 min. They were then analyzed using a flow cytometer (Beckman Coulter) and observed with a laser confocal microscope (Olympus FV1200).

### 2.13 Western blot analysis and immunohistochemistry

The COAD cells were subjected to homogenization utilizing RIPA lysis buffer supplemented with proteinase inhibitors. Total protein was isolated via 12.5% SDS-PAGE, followed by the transfer of proteins from the gels to polyvinylidene fluoride membranes. The membranes were blocked with 5% skimmed milk and then incubated overnight at 4°C with lipoic acid, FDX1, and β-actin antibodies. Following incubation with horseradish peroxidase-conjugated IgG secondary antibodies, protein expression levels were detected using ECL reagent and visualized with a chemiluminescence imager. The blots were semi-quantified by ImageJ software (1.42q, National Institutes of Health, United States). The immunohistochemical (IHC) experimental procedure involved incubating tissue sections with primary antibodies against FDX1 at 4°C overnight, followed by incubation with HRP-conjugated secondary antibodies to detect the specific binding of FDX1 through an enzymatic reaction.

### 2.14 Statistical analysis

Univariate Cox regression analysis and survival analysis were conducted using R software (version 3.6.3) to evaluate the prognostic significance of genes and generate Kaplan-Meier curves. Additionally, multivariate Cox regression analysis was performed to identify prognostic genes and establish a prognostic model. For data that followed a normal distribution, Student’s t-test and ANOVA were conducted. Each experiment was repeated three times and experimental data were analyzed and output using GraphPad Prism 10.1.2 (GraphPad Inc., San Diego, CA, United States). A *p*-value less than 0.05 was considered statistically significant.

## 3 Results

### 3.1 Expressions of CRGs in COAD tissues

**FIGURE 1:**
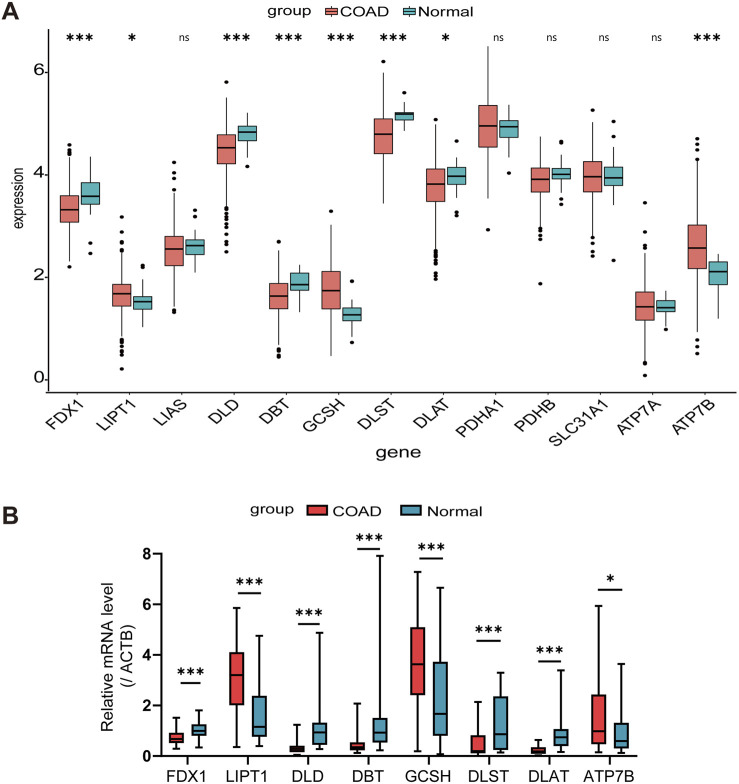
Expression levels of CRGs in COAD patients. **(A)** Comparison based on TCGA Data between normal colon tissues and COAD tissues. **(B)** Validation of TCGA analysis results through RT-qPCR detection in 30 pairs of COAD tissues and adjacent normal tissues. ^*^ *p* < 0.05, ^***^ *p* < 0.001, ^ns^not significant.

In our analysis, we collected a total of 494 samples from the TCGA database, including both normal and COAD tissues. The expressions of FDX1, LIPT1, DLD, DBT, GCSH, DLST, DLAT, and ATP7B were found to be statistically significant (Figure 1A). Through RT-qPCR validation of 30 pairs of COAD cancer and adjacent tissues from Nanfang Hospital, it was found that the expression trends of these eight CRGs in COAD cancer and adjacent tissues were consistent with the results obtained from TCGA database analysis (Figure 1B). After deleting the samples without clinical information, we compared the expression levels of the 13 CRGs across different T, N, and M stages, and used the ANOVA method to statistically analyze the gene expression levels among these stages (Supplementary Figures S1–3).

### 3.2 Functional enrichment analysis of a cuproptosis-relevant gene module derived from WGCNA

**FIGURE 2:**
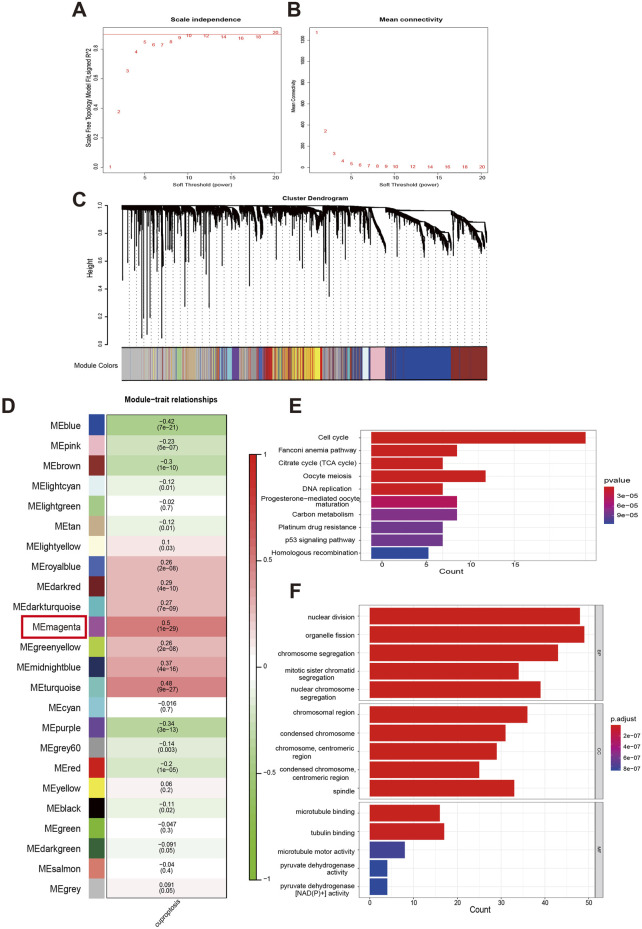
Analysis of functional enrichment in a cuproptosis-relevant gene module identified by WGCNA. **(A, B)** Distribution and trend of scale-free topology model fit and mean connectivity along with soft threshold. **(C)** The top hierarchical clustering dendrogram depicts gene clusters based on dissimilarity measurements, with color bands representing associated gene modules. **(D)** The mean correlations between modules and cuproptosis. **(E, F)** KEGG pathways and GO analysis for functional enrichment of top 100 genes in magenta module.

Next, we integrated GSVA and WGCNA to explore genes that may be associated with cuproptosis. Firstly, used GSVA to score the activity of the 13 CRGs in each COAD sample. The combination of the sample clustering dendrogram and the heatmap of GSVA scores reflected the relationships and differences among the samples (Supplementary Figure S4A). Then, we used the WGCNA algorithm to look for gene modules closely associated with cuproptosis. In WGCNA, a soft threshold of six was selected based on the scale-free model fit and mean connectivity calculations, and genes were subsequently re-clustered into fewer modules using a topological similarity matrix (Figures 2A, B). Genes were clustered using the Topological overlap matrix (TOM) and then partitioned into distinct modules. A heatmap of gene correlations based on TOM was plotted, revealing strong interactions within gene modules and relative independence between different modules (Supplementary Figure S4B). Each module was assigned a color value, which was integrated into the clustering tree to differentiate the modules (Figure 2C). We calculated the correlation and significance of the modules with cuproptosis, created a correlation heatmap, and selected the magenta module (r = 0.5, *p* < 0.001) as the most relevant based on its absolute correlation value (Figure 2D). Cluster plot analysis further confirmed that the magenta module exhibits the strongest correlation with cuproptosis (Supplementary Figure S4C). When the gene significance (GS) and module membership (MM) were plotted on a scatter plot, it was found that there had been a strong correlation between the magenta module and cuproptosis (Supplementary Figure S4D). The top 100 core genes with the highest connectivity in the magenta module were screened by MM and GS values, and their functions were revealed using KEGG and GO analyses together with the 13 CRGs we studied (Figures 2E, F). The KEGG analysis revealed that these genes are primarily involved in pathways such as the cell cycle, Fanconi anemia pathway, citrate cycle (TCA cycle), oocyte meiosis, DNA replication, progesterone-mediated oocyte maturation, carbon metabolism, platinum drug resistance, p53 signaling pathway, and homologous recombination. The GO functional analysis showed that, in terms of BP, these genes are associated with nuclear division, organelle fission, chromosome segregation, mitotic sister chromatid segregation, and nuclear chromosome segregation. For CC, they are linked to chromosomal regions, condensed chromosomes, centromeric regions, and spindles. Regarding MF, they regulate activities such as pyruvate dehydrogenase activity and pyruvate dehydrogenase [NAD(P)^+^] activity. The aforementioned research served as the foundation for our subsequent establishment of a prognostic model for COAD.

### 3.3 Establishment and validation of a novel prognostic risk model based on CRGs for COAD patients

**FIGURE 3:**
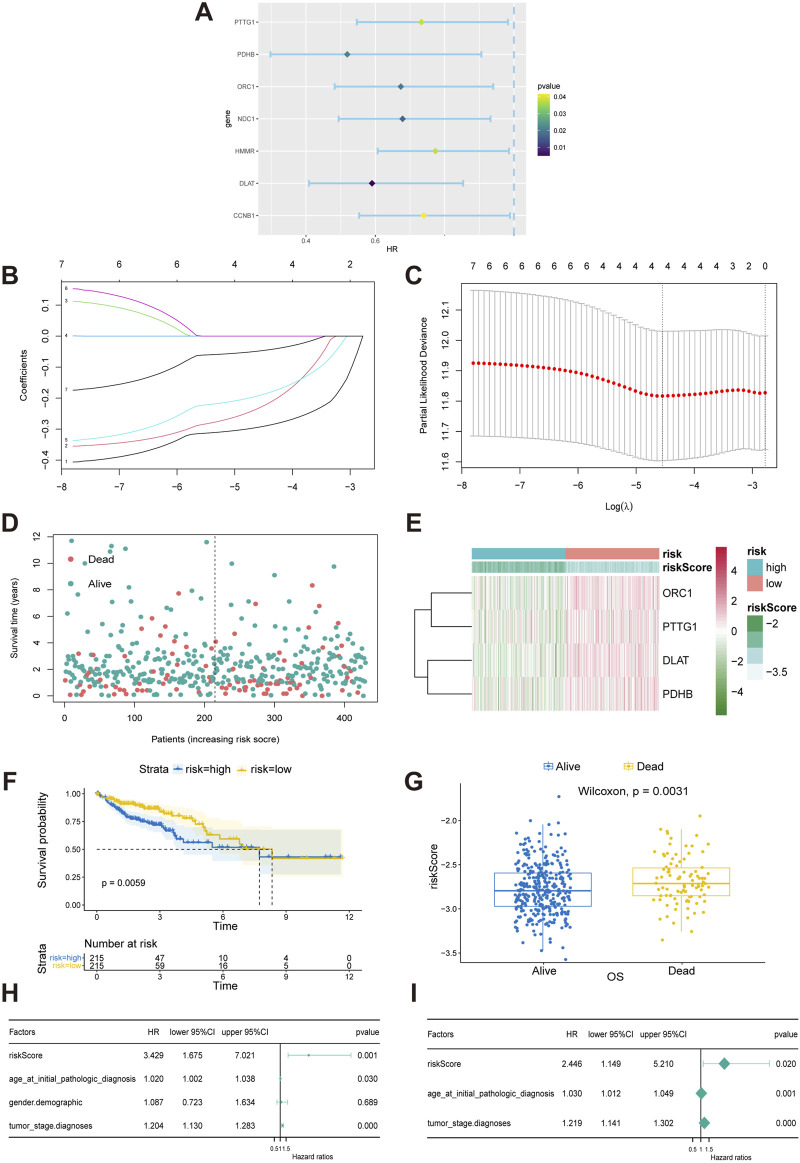
Construction and validation of a CRGs-related risk model. **(A)** Results of the univariate Cox regression analysis of prognostic genes in COAD patients. **(B, C)** Results of the LASSO regression model. **(D)** Patient stratification into high-risk and low-risk groups based on median risk score. **(E)** Heat map of four gene expression in LASSO model. **(F)** Survival differences between the high-risk and low-risk groups in TCGA-COAD patients. The table below the survival curves displays the annual number of patients alive in each year. **(G)** Risk scores for alive or dead groups. **(H, I)** Univariate and multivariate Cox regression analyses of the CRGs-related signature as an independent prognostic factor for COAD.

To explore the value of CRGs in predicting the prognosis of COAD patients, we first screened candidate prognostic CRGs using univariate Cox proportional hazards regression. Significant differences were observed in several genes, including PTTG1 (hazard ratio, HR: 0.590; 95% confidence interval, CI: 0.547–0.983, *p* = 0.038), PDHB (HR: 0.519, 95% CI: 0.297–0.906, *p* = 0.021), ORC1 (HR: 0.674, 95% CI: 0.483–0.940, *p* = 0.020), NDC1 (HR: 0.679, 95% CI: 0.494–0.932, *p* = 0.017), HMMR (HR: 0.773, 95% CI: 0.607–0.986, *p* = 0.038), NDC1 (HR: 0.679, 95% CI: 0.494–0.932, *p* = 0.017), HMMR (HR: 0.773, 95% CI: 0.607–0.986, *p* = 0.038), DLAT (HR: 0.590, 95% CI: 0.409–0.853, *p* = 0.005), and CCNB1 (HR: 0.740, 95% CI: 0.553–0.989, *p* = 0.042) (Figure 3A). Using these genes, we further performed Lasso regression analysis with ten-fold cross-validation to select the optimal λ value and identified the genes utilized in the best model as the final prognostic genes for the subsequent construction of the prognostic model (Figures 3B, C). The prognostic model used to calculate the risk score is: risk score = (−0.19 × Exp(ORC1)) + (−0.05 × Exp(PTTG1)) + (−0.29 × Exp(DLAT)) + (−0.23 × Exp(PDHB)), where Exp(·) indicates the expression level of the CRGs. Patients were then divided into high-risk and low-risk groups based on the median risk score (Figure 3D). The expression of these four CRGs in the high-risk and low-risk groups was displayed in a heatmap (Figure 3E). Risk curves indicated that patients in the low-risk group had significantly better overall survival (OS) (Figure 3F). The correlation between OS and risk score was further confirmed using the Wilcoxon test, which revealed that patients with lower scores exhibited a better prognosis (Figure 3G). Subsequent univariate and multivariate Cox regression analyses were conducted to assess the prognostic value of the model for COAD patients, revealing that the risk score derived from the model’s formula outperformed common clinicopathological characteristics in predicting patient outcomes (Figures 3H, I).

### 3.4 Differential gene expression profiles and biological function analysis between high-risk and low-risk COAD patients

**FIGURE 4:**
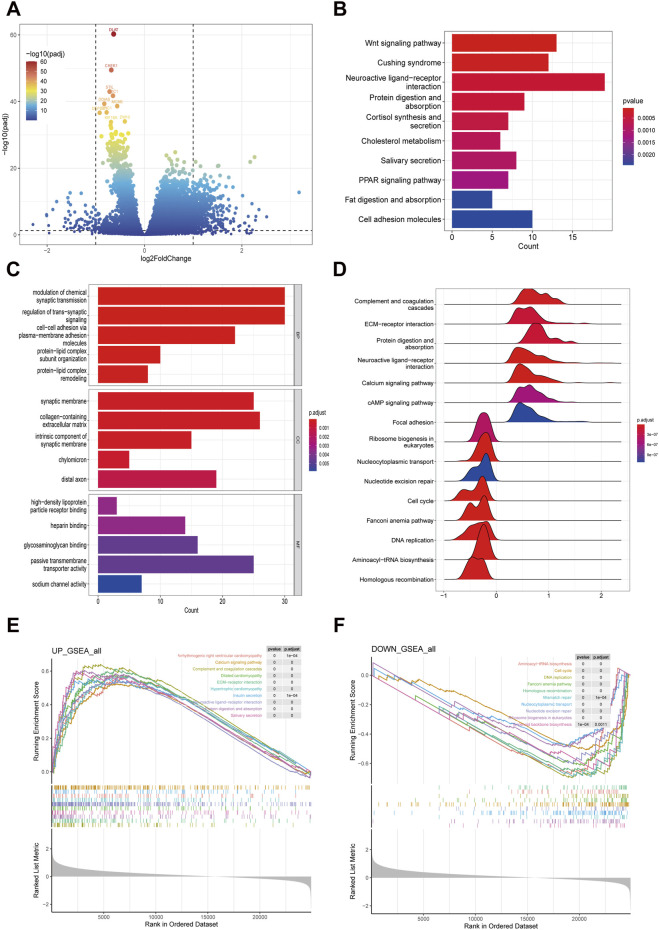
Exploring differentially expressed genes between high-risk and low-risk groups in COAD patients. **(A)** Volcano plot showing the differentially expressed genes in high- and low risk group samples. **(B, C)** Functional enrichment analyses including KEGG pathways enrichment as well as GO enrichment analysis for differentially expressed genes in high- and low risk group. **(D–F)** GSEA functional enrichment analyses for high- and low risk group.

A volcano plot displayed the DEGs between high-risk and low-risk groups in COAD patients, identified under stringent screening criteria (*p* < 0.05, |log fold change|>1), among which 490 genes were significantly upregulated and 70 genes were significantly downregulated (Figure 4A).

Subsequently, we delved into the potential biological functions and processes of these DEGs through KEGG and GO functional analyses. The KEGG analysis unveiled that the DEGs were predominantly implicated in various pathways, notably the Wnt signaling pathway, Cushing syndrome, neuroactive ligand-receptor interaction, protein digestion and absorption, cortisol synthesis and secretion, cholesterol metabolism, salivary secretion, and the PPAR signaling pathway (Figure 4B). In terms of the GO analysis, for BP, the genes contributed to the modulation of chemical synaptic transmission, regulation of trans-synaptic signaling, cell-cell adhesion via plasma-membrane adhesion molecules, protein-lipid complex subunit organization, and protein-lipid complex remodeling. For CC, there was an increase mainly in high-density lipoprotein particle receptor binding, heparin binding, glycosaminoglycan binding, passive transmembrane transporter activity, and sodium channel activity. For MF, the genes primarily regulated the synaptic membrane, collagen-containing extracellular matrix, intrinsic component of synaptic membrane, chylomicron, and distal axon (Figure 4C).

To further elucidate the functions of the DEGs, we employed GSEA. The analysis revealed that the following pathways were upregulated in the hish-risk group: Arrhythmogenic right ventricular cardiomyopathy, Calcium signaling pathway, Complement and coagulation cascades, Dilated cardiomyopathy, ECM-receptor interaction, Hypertrophic cardiomyopathy, Insulin secretion, Neuroactive ligand-receptor interaction, Protein digestion and absorption, and Salivary secretion. Conversely, the downregulated pathways included: Aminoacyl-tRNA biosynthesis, Cell cycle, DNA replication, Fanconi anemia pathway, Homologous recombination, Mismatch repair, Nucleocytoplasmic transport, Nucleotide excision repair, Ribosome biogenesis in eukaryotes, and Terpenoid backbone biosynthesis (Figures 4D–F).

### 3.5 Differences in the tumor microenvironment between high-risk and low-risk COAD patients

**FIGURE 5:**
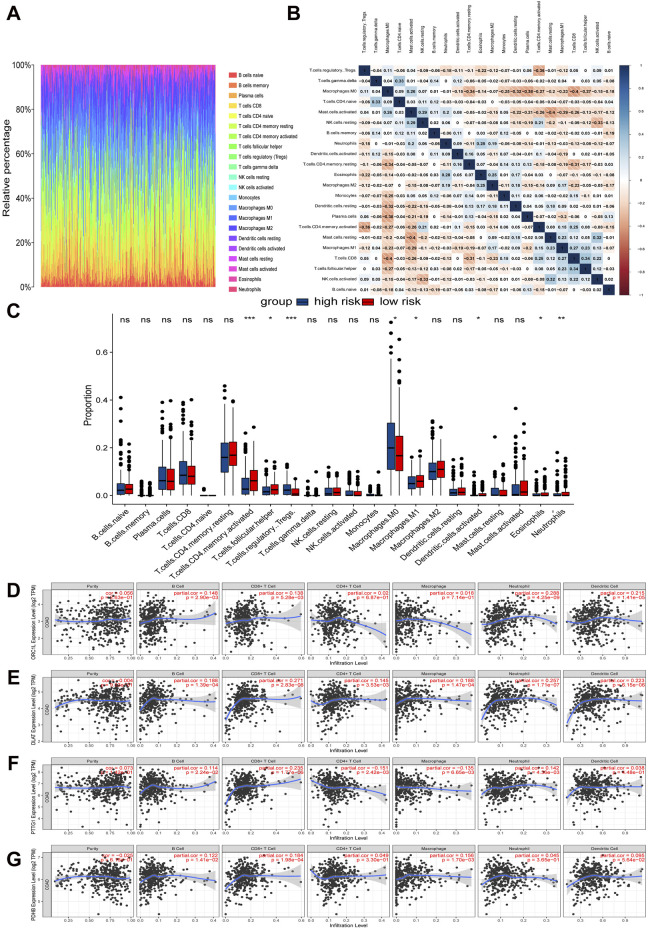
Relationships between expression of cuproptosis-related signature and tumor immune infiltrations. **(A)** Relative percentages of immune cell types in TCGA-COAD samples. **(B)** Heat map of correlation between immune cell subtypes. **(C)** Differences in immune cell infiltration proportions between high-risk and low-risk groups. **(D–G)** Correlation between four hub CRGs in the risk model and immune cell subtypes.

We used the CIBERSORT algorithm to obtain the infiltration proportions of immune cells in each TCGA-COAD sample and presented them in the form of a heatmap (Figure 5A). Another heatmap was generated to illustrate the correlations between various immune cell types (Figure 5B). Additionally, the resulting bar plot visualized the differences in the infiltration proportions of 22 immune cell types between the high-risk group and the low-risk group (Figure 5C). Notably, the low-risk subtype exhibited increased infiltration of activated CD4^+^ memory T cells, follicular helper T (Tfh) Cells, M1 macrophages, activated dendritic cells, eosinophils, and neutrophils. Conversely, the high-risk subtype showed higher levels of regulatory T cells (Tregs) and M0 macrophages.

The further analysis revealed that the infiltration of immune cells was correlated with the expression of four key CRGs in the prognostic risk model (Figures 5D–G). Specifically, the expression of ORC1 was positively correlated with B cells, CD8^+^ T cells, neutrophils, and dendritic cells. DLAT exhibited a positive correlation with B cells, CD8^+^ T cells, CD4^+^ T cells, macrophages, neutrophils, and dendritic cells. PTTG1 demonstrated a positive correlation with B cells, CD8^+^ T cells, and neutrophils, while it showed a negative correlation with CD4^+^ T cells and macrophages. Lastly, PDHB was positively correlated with B cells, CD8^+^ T cells, and macrophages.

Subsequently, we utilized the ESTIMATE algorithm to assess tumor purity by calculating StromalScore, ImmuneScore, and ESTIMATEScore (Supplementary Figures S5A–F). The results revealed a significant positive correlation between risk scores and StromalScores (r = 0.15, *p* = 0.0019). In contrast, no statistically significant correlation was observed between risk scores and ImmuneScores (r = 0.045, *p* = 0.35). Overall, ESTIMATEScores, which are the sum of StromalScores and ImmuneScores, showed a weak but statistically significant correlation with risk scores (r = 0.11, *p* = 0.027).

### 3.6 Mutation landscape and immune characteristics comparison of high-risk and low-risk COAD patients

**FIGURE 6:**
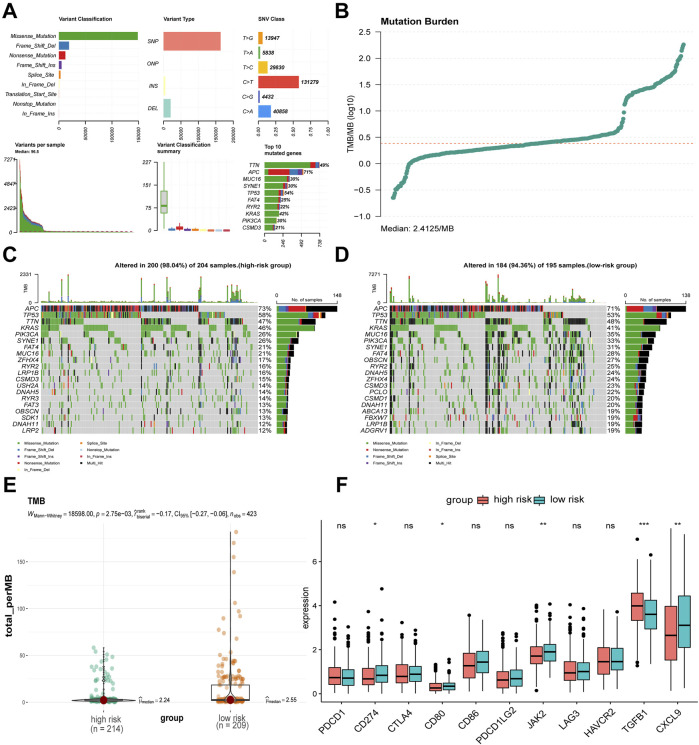
Mutation, TMB, and immune checkpoints differences between risk groups. **(A, B)** Mutation landscape of TCGA-COAD. **(C, D)** Differences in somatic single nucleotide mutations between high-risk and low-risk groups. **(E)** Difference in TMB values between high-risk and low-risk groups. **(F)** Differences in immune checkpoint related gene expression between high-risk and low-risk groups. ^*^ *p* < 0.05, ^**^ *p* < 0.01, ^***^ *p* < 0.001, ^ns^not significant.

By analyzing the mutation characteristics of COAD data from TCGA, we gained insights into various aspects, including variant classification, variant type, single nucleotide variants (SNVs), the number of variants per sample, an overview of the different types of variants, the top 10 mutated genes, and the tumor mutation burden (TMB) (Figures 6A, B). Our analysis revealed that APC, TP53, TTN, and KRAS had the highest mutation proportions in both the high-risk and low-risk groups. Notably, the proportion of mutations in APC, TP53, and KRAS was higher in the high-risk group compared to the low-risk group (Figures 6C, D). The TMB level of the low-risk group was significantly higher than that in the high-risk group (Figure 6E). To further investigate, we conducted a Wilcoxon rank-sum test to compare the expression levels of immune checkpoints between the two risk groups. The results showed that CD274 (also known as PD-L1), CD80, JAK2, and CXCL9 exhibited lower expression, while TGFβ1 exhibited higher expression in the high-risk group compared to the low-risk group (Figure 6F). Finally, we assessed CYT, a measure of tumor-infiltrating T lymphocyte activity and a prognostic indicator, in both groups. Although there was no statistically significant difference in CYT between the high- and low-risk groups, the high-risk group had numerically lower CYT scores than the low-risk group (Supplementary Figures S6).

### 3.7 FDX1 has an inhibitory effect on colon adenocarcinoma

The *Science* article highlights the pivotal role of FDX1 in inducing cuproptosis (Tsvetkov et al., 2022). However, based on our aforementioned analysis, both TCGA database and clinical samples suggest that the low expression of FDX1 in COAD tissues may promote tumor progression by attenuating the cuproptosis effect induced by ES. This finding raises a critical scientific question: As a key CRG, does the loss of FDX1 expression directly contribute to driving the development and progression of COAD? To explore this issue, we conducted a series of experiments to investigate the therapeutic potential of FDX1 in COAD. Our survival analysis revealed a significant association between high FDX1 expression and better OS in COAD patients (*p* = 0.019) (Supplementary Figure S7A). To further validate this finding, we measured FDX1 mRNA levels in cancer and adjacent tissues from 30 COAD patients at Nanfang Hospital using RT-qPCR. The results showed that FDX1 expression was significantly downregulated in cancer tissues compared to adjacent tissues (Supplementary Figure S7B). This was further confirmed by immunohistochemical analysis, which also demonstrated low FDX1 expression in cancer tissue (Supplementary Figure S7C).

**FIGURE 7:**
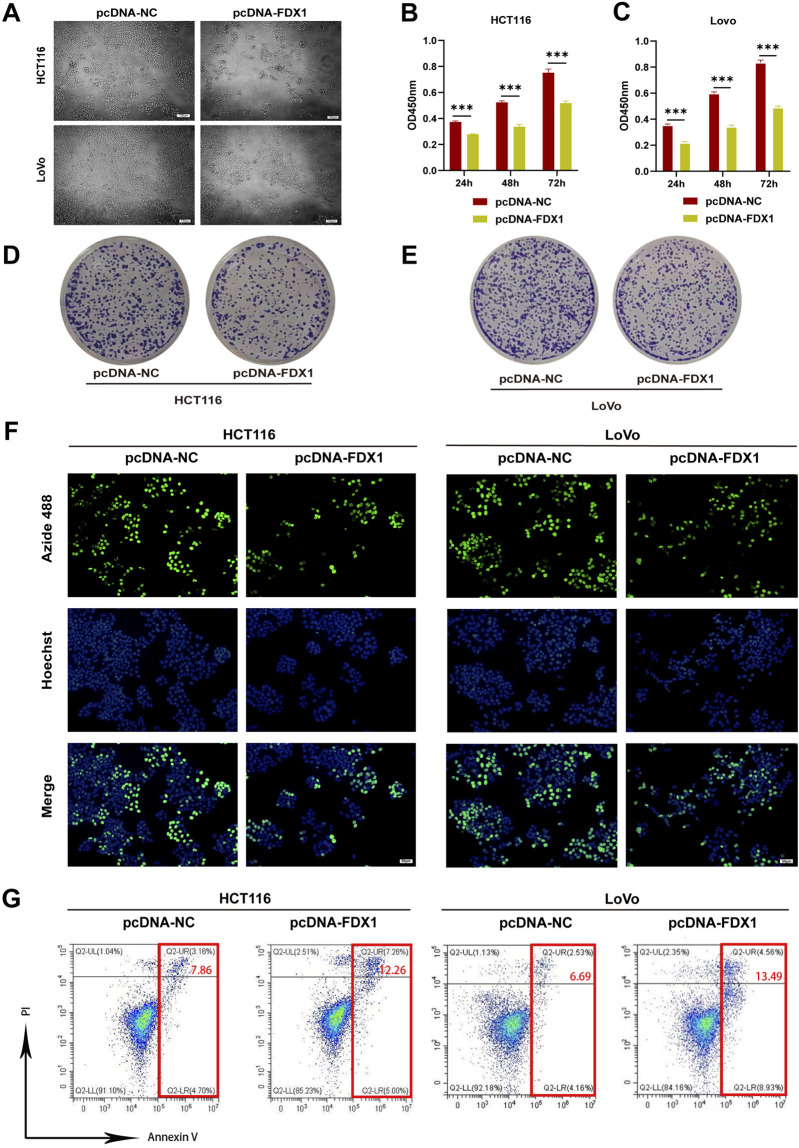
Effect of FDX1 overexpression on the proliferation and apoptosis levels of COAD cells. **(A)** Morphological changes in HCT116 and LoVo cells after overexpressing FDX1. Scale bar is set at 100 μm. **(B, C)** CCK-8 assay evaluated proliferation rates of HCT116 and LoVo cells after overexpressing FDX1. **(D, E)** Colony formation assay of HCT116 and LoVo cells after overexpressing FDX1. **(F)** EdU proliferation assay assessed proliferation levels of HCT116 and LoVo cells after overexpressing FDX1. **(G)** Flow cytometry analysis of apoptosis levels in HCT116 and LoVo cells after overexpressing FDX1. ^***^ *p* < 0.001.

Next, we utilized HCT116 and LoVo, two types of COAD cells, to verify the effect of FDX1 on COAD through *in vitro* experiments. Observation of the morphology of HCT116 and LoVo cells revealed that overexpression of FDX1 led to a decrease in the total cell count and an increase in the number of rounded, apoptotic cells (Figure 7A). Additionally, CCK-8 assays showed that FDX1 overexpression significantly inhibited the proliferation of these cells at 24, 48, and 72 h (Figures 7B, C). Clone formation assays confirmed that FDX1 overexpression reduced the number of cell colonies formed by HCT116 and LoVo cells (Figures 7D, E; Supplementary Figures S8A, B). The EdU proliferation assay also indicated that FDX1 overexpression significantly decreased the proliferation rate of these cells, as evidenced by reduced fluorescence intensities (Figure 7F; Supplementary Figures S8C, D). Finally, flow cytometry analysis with Annexin V/PI double staining showed that FDX1 overexpression significantly increased the levels of apoptosis in HCT116 and LoVo cells (Figure 7G; Supplementary Figures S8E, F). These findings collectively suggest that FDX1 has an inhibitory effect on COAD cell growth and proliferation.

### 3.8 FDX1 promotes elesclomol-induced cuproptosis in colon adenocarcinoma cells

**FIGURE 8:**
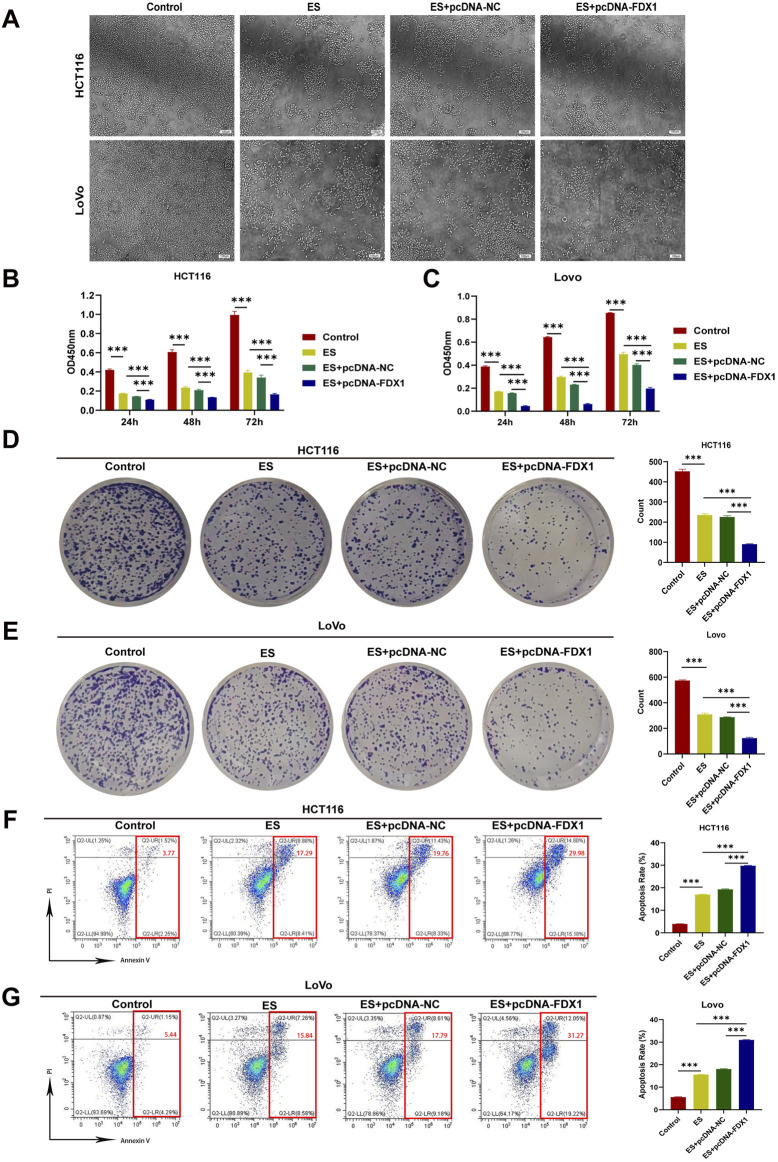
Effect of elesclomol treatment combined with FDX1 overexpression on the proliferation and apoptosis levels of COAD cells. **(A)** Morphological changes in HCT116 and LoVo cells after overexpression of FDX1. Scale bar is set at 100 μm. **(B, C)** CCK-8 assay evaluated proliferation rates of HCT116 and LoVo cells in Control, ES, ES + pcDNA-NC, and ES + pcDNA-FDX1 groups. **(D, E)** Colony formation assay of HCT116 and LoVo cells in Control, ES, ES + pcDNA-NC, and ES + pcDNA-FDX1 groups. **(F, G)** Flow cytometry analysis of apoptosis levels in HCT116 and LoVo cells from Control, ES, ES + pcDNA-NC, and ES + pcDNA-FDX1 groups. ^***^ *p* < 0.001.

**FIGURE 9:**
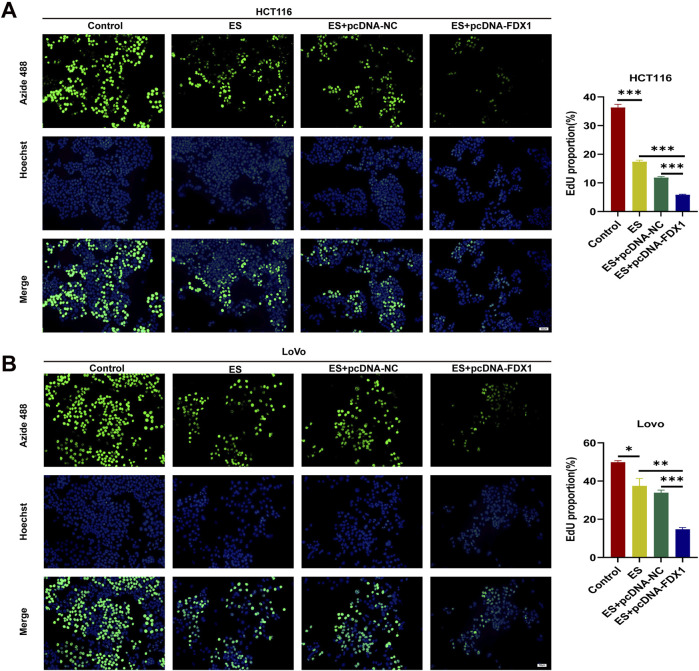
**(A, B)** EdU proliferation assay with HCT116 and LoVo cells in Control, ES, ES + pcDNA-NC, and ES + pcDNA-FDX1 groups. Scale bar is set at 50 μm ^*^ *p* < 0.05, ^**^ *p* < 0.01, ^***^ *p* < 0.001.

**FIGURE 10:**
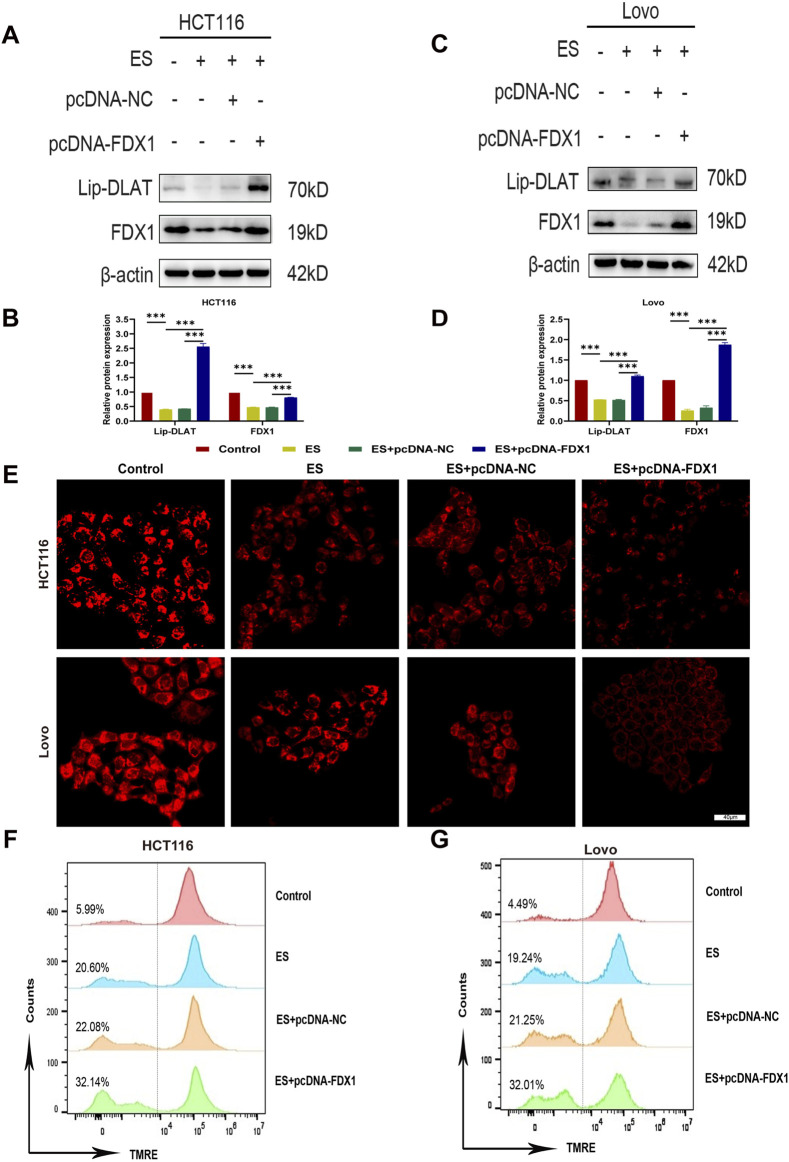
Effect of elesclomol combined with FDX1 overexpression on cuproptosis levels in COAD cells. **(A–D)** Western blot analysis of lipoylated DLAT (lip-DLAT) and FDX1 expression of HCT116 and LoVo cells in the Control, ES, ES + pcDNA-NC, and ES + pcDNA-FDX1 groups. **(E)** The fluorescence intensity of HCT116 and LoVo cells in Control, ES, ES + pcDNA-NC, and ES + pcDNA-FDX1 groups was observed using confocal microscopy after staining with the mitochondrial dye TMRE. Scale bar is set at 40 μm. **(F, G)** Flow cytometry analysis of mitochondrial membrane potential levels in HCT116 and LoVo cells after TMRE staining in Control, ES, ES + pcDNA-NC, and ES + pcDNA-FDX1 groups. ^***^ *p* < 0.001.

To address the research gap concerning the combined effects of FDX1 and elesclomol (ES) in the treatment of COAD, we further investigated whether FDX1 enhances ES-induced cuproptosis in COAD cells. We divided HCT116 and LoVo cells each into four treatment groups: the ES group, which received a 2-h pulse treatment with 100 nM ES and 2 μM CuCl_2_; the ES + pcDNA-NC group, which received empty pcDNA plasmids in addition to the ES treatment; the ES + pcDNA-FDX1 group, which received FDX1 plasmids in addition to the ES treatment; and a control group that received no treatment. The morphological examination showed a reduction in cell counts for both HCT116 and LoVo cells, along with an increase in the proportion of rounded cells in the ES group. Notably, the ES + pcDNA-FDX1 group showed a further decrease in cell counts and increase in the proportion of rounded cells compared to both the ES and ES + pcDNA-NC groups (Figure 8A). Consistent with these findings, CCK-8 assays, clone formation assays, and EdU proliferation assays demonstrated significantly inhibited proliferation in the ES + pcDNA-FDX1 group compared to the other treatment groups (Figures 8B–E, 9A, B). Flow cytometry analysis with Annexin V/PI double staining confirmed a significant increase in apoptosis levels in the ES + pcDNA-FDX1 group (Figures 8F, G). Western blot analysis revealed that ES treatment reduced the protein expressions of FDX1 and lipoylated DLAT (Lip-DLAT). However, combined treatment with ES and FDX1 overexpression led to a significant increase in these protein levels (Figures 10A–D). To assess mitochondrial membrane potential, HCT116 and LoVo cells were stained with the mitochondrial dye TMRE and analyzed using laser confocal microscopy and flow cytometry. The results showed a significant reduction in TMRE fluorescence intensity in the ES + pcDNA-FDX1 group compared to the ES and ES + pcDNA-NC groups (Figure 10E; Supplementary Figures S8A, B), and flow cytometry analysis further confirmed a higher proportion of cells with decreased TMRE expression in the ES + pcDNA-FDX1 group (Figures 10F, G; Supplementary Figures S8C, D). To verify whether FDX1 activates ES-induced cuproptosis rather than other forms of cell death, COAD cells were treated with a 2-h pulse of 100 nM and 2 μM CuCl2 in combination with 20 μM of the cuproptosis inhibitor TTM. The results showed that the addition of TTM significantly reversed the inhibitory effect of ES combined with FDX1 overexpression on COAD cell proliferation and its promotional effect on apoptosis (Supplementary Figure S10), further demonstrating that FDX1 can enhance ES-induced cuproptosis.

Collectively, these results indicate that FDX1 overexpression in COAD cells during ES treatment enhances the reduction in cell proliferation, increases apoptosis, increases lipoylation levels of DLAT, and decreases mitochondrial membrane potential compared to ES treatment alone. This suggests a synergistic effect between FDX1 and ES, promoting mitochondrial dysfunction and cuproptosis in COAD cells.

## 4 Discussion

Cuproptosis, a recently discovered cell death mechanism, has garnered significant attention in cancer research—particularly in prognostic evaluation, molecular classification, and therapeutic strategy development—due to advances in cancer genomics databases and computational biology (Huang et al., 2022; Li et al., 2022; Mi et al., 2023; Shen et al., 2023). Despite various treatments like surgery, chemotherapy, radiotherapy, and immunotherapy, the overall survival rate for COAD patients remains unsatisfactory. This is largely attributed to the complex pathogenesis and diverse biological characteristics of COAD, which hinder precise diagnosis and effective prognostic marker identification (Qaderi et al., 2020; Xie et al., 2023). Current research on the role of CRGs in COAD is insufficient, highlighting the need to assess their prognostic and therapeutic value. Building on our previous study that established a combined risk score model for prognostic assessment of soft tissue sarcoma patients based on CRGs—providing new insights into prognostic biomarker screening by exploring CRG-immunity interactions (Li et al., 2022)—we aim to apply this approach to COAD. Leveraging this experience, we seek to develop precise diagnostic tools and effective prognostic markers for COAD by investigating the role of CRGs, thereby offering new strategies for patient assessment and treatment.

Certain studies have demonstrated differences in CRGs between tumor and normal tissues (Bian et al., 2022; Peng et al., 2022). Through the analysis of COAD samples in the TCGA database, we identified significant expression differences between tumor tissues and normal tissues for eight CRGs. These differences were further validated using RT-qPCR to ensure the accuracy of the results. The study revealed that, compared to normal tissues, genes FDX1, DLD, DBT, DLST, and DLAT were significantly downregulated in colorectal cancer tissues, while LIPT1, GCSH, and ATP7B genes were upregulated. FDX1 (ferredoxin 1), as a core regulator of cuproptosis, is closely associated with tumor cell activity and immune response (Wang et al., 2023). Studies have shown that FDX1 can inhibit the growth and progression of colorectal cancer by delaying the epithelial-mesenchymal transition (EMT) process (Wang et al., 2025), and its low expression is significantly correlated with poor prognosis in colorectal cancer patients ( Wang et al., 2023). DLD (dihydrolipoamide dehydrogenase), as the E3 component of three α-ketoacid dehydrogenase complexes (Brautigam et al., 2006), is a key gene regulating cuproptosis. Its expression level is closely related to cancer patient survival rates, immune cell infiltration, and tumor mutation status (Yang et al., 2023). It has been confirmed as a prognostic marker for various malignant tumors and plays a protective role in colorectal cancer (Lin et al., 2023). DBT (dihydrolipoamide branched-chain transacylase E2) encodes an essential component of the branched-chain α-ketoacid dehydrogenase complex (Chuang et al., 2006), and its low expression in colorectal cancer inhibits the cuproptosis process. DLST (dihydrolipoamide succinyltransferase) can bind to excess copper ions in cells, leading to the oligomerization of lipoylated DLST and inducing cell death. Studies have confirmed that the low expression of DLST is significantly associated with lower survival rates in colorectal cancer patients (Yang et al., 2023). DLAT, as a key component of the pyruvate dehydrogenase complex, participates in the tricarboxylic acid cycle and is one of the important genes regulating cuproptosis. Copper ion overload can cause oligomerization of lipoylated DLAT, thereby inducing cell death. DLAT plays a protective role in colorectal cancer, and its low expression is associated with poor prognosis in patients (Chu et al., 2023). Notably, the expression level of LIPT1 (lipoyltransferase 1) is positively correlated with the risk score of colorectal cancer (Li et al., 2024). The high expression of GCSH (γ-glutamylcysteine synthetase) has been confirmed to be associated with poor prognosis in endometrial cancer (Zhao et al., 2024) and cholangiocarcinoma (He et al., 2024) patients. The copper efflux transporter encoded by the ATP7B gene is highly expressed in colorectal cancer, and its elevated expression level is significantly correlated with poor prognosis in colorectal cancer patients receiving oxaliplatin-based chemotherapy (Martinez-Balibrea et al., 2009).

In order to uncover potential biological significance, we employed the GSVA and WGCNA method which can highly effective in processing large-scale gene data and cluster genes with similar expression patterns. WGCNA, with its unsupervised network construction strategy, is particularly suitable for exploring the molecular mechanisms of complex diseases and developing multi-gene biomarkers. Its modular output format enhances the interpretability of results, making it an essential tool in systems biology research (Langfelder et al., 2008). GSVA bridges the gap between “gene modules” and “biological functions” for WGCNA. By quantifying pathway activity, it not only enhances the interpretability of results but also untangles the regulatory mechanisms behind gene co-expression (Gu et al., 2024). Hence, our analysis bears greater innovativeness and rationality. Through GSVA and WGCNA, we successfully clustered numerous genes and identified the magenta module that is highly correlated with cuproptosis. Furthermore, we conducted comprehensive functional annotations on the top 100 genes within the magenta module using KEGG and GO analyses. The results indicate that these genes are primarily involved in cell cycle, DNA replication, platinum drug resistance, p53 signaling pathway, TCA cycle, and pyruvate dehydrogenase [NAD(P)^+^] activity. Our research aligns with previous studies showing that copper induces DNA damage repair and drug resistance (Jin et al., 2022), stabilizes p53 to cause cell cycle arrest and apoptosis in cisplatin-resistant neuroblastoma cells (Zhang et al., 2008), and affects mitochondrial respiration by targeting the pyruvate dehydrogenase complex in the TCA cycle of tumor cells undergoing cuproptosis (Tsvetkov et al., 2022). The results from KEGG and GO analyses confirm the close association between the genes we have identified and cuproptosis. This lays a solid foundation for our subsequent efforts to construct a prognostic model based on these genes and evaluate the prognostic value of CRGs in patients with COAD.

CRGs have been validated for prognostic prediction and treatment guidance in patients with various cancers (Huang et al., 2022; Li et al., 2022; Lian et al., 2023; Shen et al., 2023; Zhang et al., 2022). In this study, for the purpose of prognostic evaluation using CRGs, we employed LASSO regression, a method known for its efficient dimensionality reduction and high predictive accuracy through the selection of key features and construction of a risk score (Laing et al., 2022). By developing a novel risk score formula that incorporates the expression levels of four CRGs—ORC1, PTTG1, DLAT, and PDHB—we stratified patients into high-risk and low-risk groups, demonstrating a significant correlation between overall survival and the risk score, with better prognosis observed in the low-risk group. To explore the reasons for the poor prognosis of high-risk patients, we conducted a series of analyses on the DEGs between the high-risk and low-risk groups. Through KEGG analysis, we found that these DEGs are enriched in the Wnt signaling pathway, suggesting that abnormal activation of the Wnt signaling pathway may also weaken the sensitivity of cancer cells to cuproptosis by regulating copper ion homeostasis. A study have shown that abnormal activation of the Wnt/β-catenin signal promotes the expression of ATP7B through the β-catenin/TCF4 transcriptional complex (Liu et al., 2024). ATP7B confers resistance to cuproptosis in cancer stem cells by reducing intracellular copper content and inhibiting cuproptosis. This suggests that inhibiting the Wnt/β-catenin signaling pathway can increase the sensitivity of cancer stem cells to cuproptosis, providing a precision medicine strategy for cancer treatment. Additionally, GSEA analysis of DEGs in high-risk and low-risk COAD patients revealed that the cell cycle and DNA replication pathways are significantly suppressed in the high-risk group. As mentioned in Part 3.2, cuproptosis is associated with cell cycle and DNA replication pathways, indicating that patients in the high-risk group may be in a state of resistance to cuproptosis. We further analyzed the differences in immune condition between high-risk and low-risk COAD patients from three aspects: tumor microenvironment, tumor mutation burden, and cytolytic activity. The tumor microenvironment, comprising tumor, immune, and stromal cells interacting via chemokines and cytokines, plays a key role in cancer progression and treatment response, with immune-favorable TME subtypes enhancing immunotherapy benefits (Bagaev et al., 2021; Mellman et al., 2023). Studies reveal a close link between cuproptosis-related genes and the tumor microenvironment, where gene expressions such as SLC31A1 correlate with immune cell infiltrates, and high DLAT expression inversely relates to survival in HCC patients with high immune cell infiltration (Lian et al., 2023; Zhang et al., 2023). A CRG-based risk model suggests low-risk patients have enhanced immunogenicity and improved immunotherapy response (Zhu et al., 2022). Our CIBERSORT analysis showed higher immune cell infiltration in the low-risk group, especially CD4 memory T cells, activated dendritic cells, and neutrophils. These results support previous research and the risk model’s reliability in predicting prognosis, emphasizing the importance of immune microenvironment modification in COAD for improving patient survival. TMB, quantifying the number of mutations in tumor cells, is crucial in tumor treatment and prognosis, with a high TMB indicating more mutations, facilitating immune detection and attack, and thus improving immunotherapy response (Budczies et al., 2024). Our study revealed that the TMB level in the low-risk group was higher than in the high-risk group, suggesting greater sensitivity to immunotherapy and further supporting the previous conclusion of significantly higher immune cell infiltration in the low-risk group. CYT, calculated as the geometric mean of GZMA and PRF1 mRNA expression in tissue, generally indicates a stronger anti-tumor immune response and heightened sensitivity to immune checkpoint inhibitors, reflecting the host’s immune status (Rooney et al., 2015). Our study demonstrated that the CYT level was higher in the low-risk group compared to the high-risk group, aligning with previous research, although the lack of statistical significance may be due to large individual differences or an insufficient sample size. Overall, the novel risk model we established based on CRGs effectively predicts the immune status of COAD patients and provides a valuable assessment for determining their sensitivity to immunotherapy.

Given the promising role of CRGs in predicting the prognosis of COAD patients, their potential therapeutic applications in treating COAD patients are worth further exploration. In different cancer cell lines, cuproptosis is primarily driven by mitochondrial protein toxic stress mediated by FDX1 (Tsvetkov et al., 2022; 2019), making FDX1 a key CRG for utilizing cuproptosis to eliminate tumor cells. FDX1 has been shown to inhibit multiple types of cancer. In non-small cell lung cancer, METTL3 may promote the development of the disease by facilitating the maturation of pri-miR-21-5p, upregulating miR-21-5p, and targeting the inhibition of FDX1 (Qian et al., 2023). According to Ran et al. (Ran et al., 2023), FDX1 is a critical CRG in uterine corpus endometrial carcinoma, enhancing immune infiltration and attenuating proliferation and metabolism. In hepatocellular carcinoma, the downregulation of FDX1 contributes to metabolic reprogramming, leading to reactive oxygen species-mediated mitochondrial autophagy and activation of the PI3K/AKT signaling pathway, resulting in poor prognosis (Sun et al., 2024). In renal clear cell carcinoma, reduced FDX1 expression is associated with disease progression, poor prognosis, and dysregulation of immune cell infiltration (Wang et al., 2022). The curcumin analog PBPD induces cuproptosis and endoplasmic reticulum stress in cervical cancer cells via the Notch1/RBP-J/NRF2/FDX1 pathway (Zhang et al., 2024). In gastric cancer, the lactylation of METTL16 promotes cuproptosis through m6A modification on FDX1 mRNA (Sun et al., 2023). Our research conclusions are consistent with these studies, indicating that FDX1 plays an inhibitory role in the initiation and progression of tumor cells. We also observed that, compared to normal tissues, the expression of FDX1 is significantly reduced in COAD tissues, and patients with high FDX1 expression have a better prognosis. Both of these findings are consistent with the conclusions of a previous study (L. Wang et al., 2023).

FDX1 is the direct target of the copper ionophore elesclomol (Tsvetkov et al., 2019), and it plays a crucial role in the release of Cu^+^ by elesclomol (Zulkifli et al., 2023). Elesclomol possesses a unique ability to deliver copper into mitochondria, thereby inducing copper-dependent cell death in tumor cells (Tsvetkov et al., 2022). Before elesclomol was discovered to induce cuproptosis to eliminate tumor cells, it had already been demonstrated to trigger copper-dependent apoptosis or ferroptosis in melanoma, leukemia, or CRC cells by promoting the generation of ROS or facilitating the degradation of ATP7A in the context of anti-tumor therapy (Gao et al., 2021; Kirshner et al., 2008). In a randomized Phase II study, the addition of elesclomol to paclitaxel significantly reduced the risk of disease progression or death in patients with melanoma (O'Day et al., 2009). Research conclusions on FDX1 and COAD indicate that: 4-OI inhibits aerobic glycolysis by targeting GAPDH to promote elesclomol-induced cuproptosis in colorectal cancer (Yang et al., 2023; Wang et al., 2023); FDX1 expression is significantly lower in COAD than in normal tissues (Su and Zhang, 2023); FDX1 suppresses the growth and progression of colorectal cancer by retarding EMT progression (Wang et al., 2025); high FDX1 expression correlates with favorable prognosis and immune cell infiltration in COAD (L. Wang et al., 2023); and knocking down FDX1 inhibits colorectal cancer progression and regulates cuproptosis by mediating the Hippo signaling pathway (Hu et al., 2025). Different from these studies, to further enhance elesclomol-induced cuproptosis and maximize its antitumor effect, we conducted the first study combining elesclomol with FDX1 overexpression in COAD cells. By examining changes in mitochondrial membrane potential and lipoylation levels of the DLAT protein, we elucidated the role of FDX1 in inducing cuproptosis in COAD. Compared to elesclomol treatment alone, this combination significantly increased the lipoylation level of DLAT, which further induced cuproptosis, leading to a more pronounced decrease in mitochondrial membrane potential, greater inhibition of cell proliferation, and enhanced apoptosis. These findings suggest that the combined use of elesclomol and FDX1 overexpression holds great promise in the treatment of COAD, filling the research gap regarding the enhancement of cuproptosis in COAD through the combination of FDX1 and elesclomol. Therefore, subsequent clinical trials should be conducted to evaluate the efficacy and safety of this combination. With advancing research and technological progress, this combinatorial treatment strategy is expected to bring new therapeutic hope to COAD patients.

Our study has several limitations. First, although the results were validated by RT-qPCR, experiments to confirm immune cell infiltration were not conducted, necessitating further investigation. Second, there is a lack of immunotherapy data for COAD patients, and broader clinical data to validate our bioinformatics findings are also missing; therefore, the predictive risk score for COAD needs further validation. Lastly, the therapeutic efficacy of FDX1 overexpression, both alone and in combination with elesclomol, for the treatment of COAD has not yet been validated in animal models or clinical trials.

## 5 Conclusion

In summary, we identified CRGs with significant prognostic value in COAD and developed a novel risk score incorporating the expression levels of ORC1, PTTG1, DLAT, and PDHB. This risk score effectively stratified patients into high-risk and low-risk groups, correlating lower risk scores with better overall survival and enhanced immune characteristics. We also demonstrated that overexpression of FDX1, combined with the copper ionophore elesclomol, significantly enhances cuproptosis and exhibits greater antitumor effects in COAD cells compared to elesclomol alone. These findings suggest that targeting cuproptosis pathways, particularly through FDX1 and elesclomol, offers a promising therapeutic strategy for COAD patients. Future studies should validate these results clinically to improve diagnostic precision and treatment outcomes.

## Data Availability

The original contributions presented in the study are included in the article/Supplementary Material, further inquiries can be directed to the corresponding authors.
