# Supplementary material for: Assessing the prognostic and therapeutic value of cuproptosis-related genes in colon adenocarcinoma patients
Source: Front Cell Dev Biol. 2025 Apr 10;13:1550982. doi: 10.3389/fcell.2025.1550982 (PMC12018357; doi:10.3389/fcell.2025.1550982)
Supplement: Supplementary file 1 [file DataSheet1.docx]

Supplementary Material

# Supplementary Figures and Tables

## Supplementary Figures


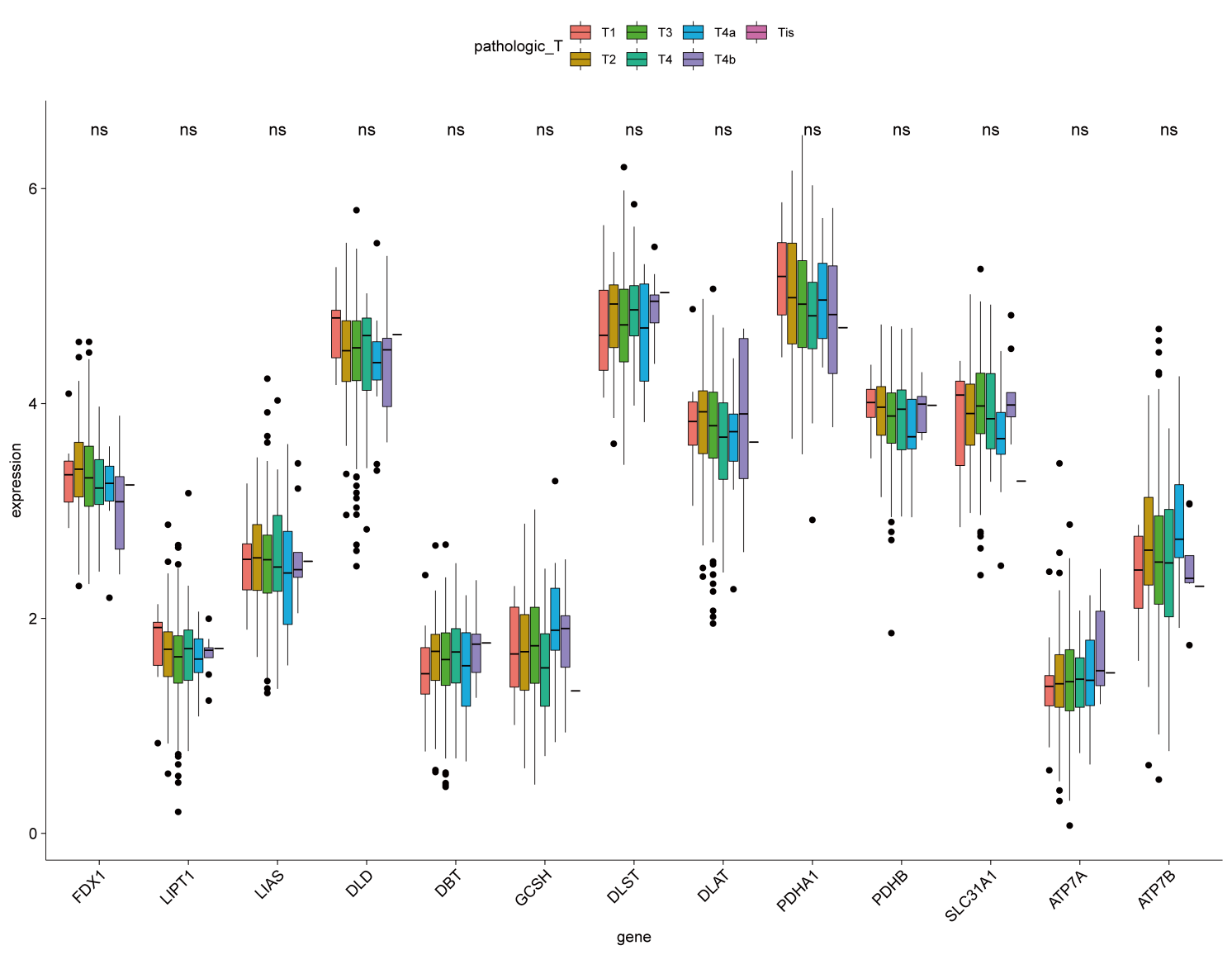


**Supplementary Figure S1.** The expression level of CRGs at different Tumor stages in COAD. ^*^*p*<0.05, ^**^*p*<0.01, ^ns^not significant.

**
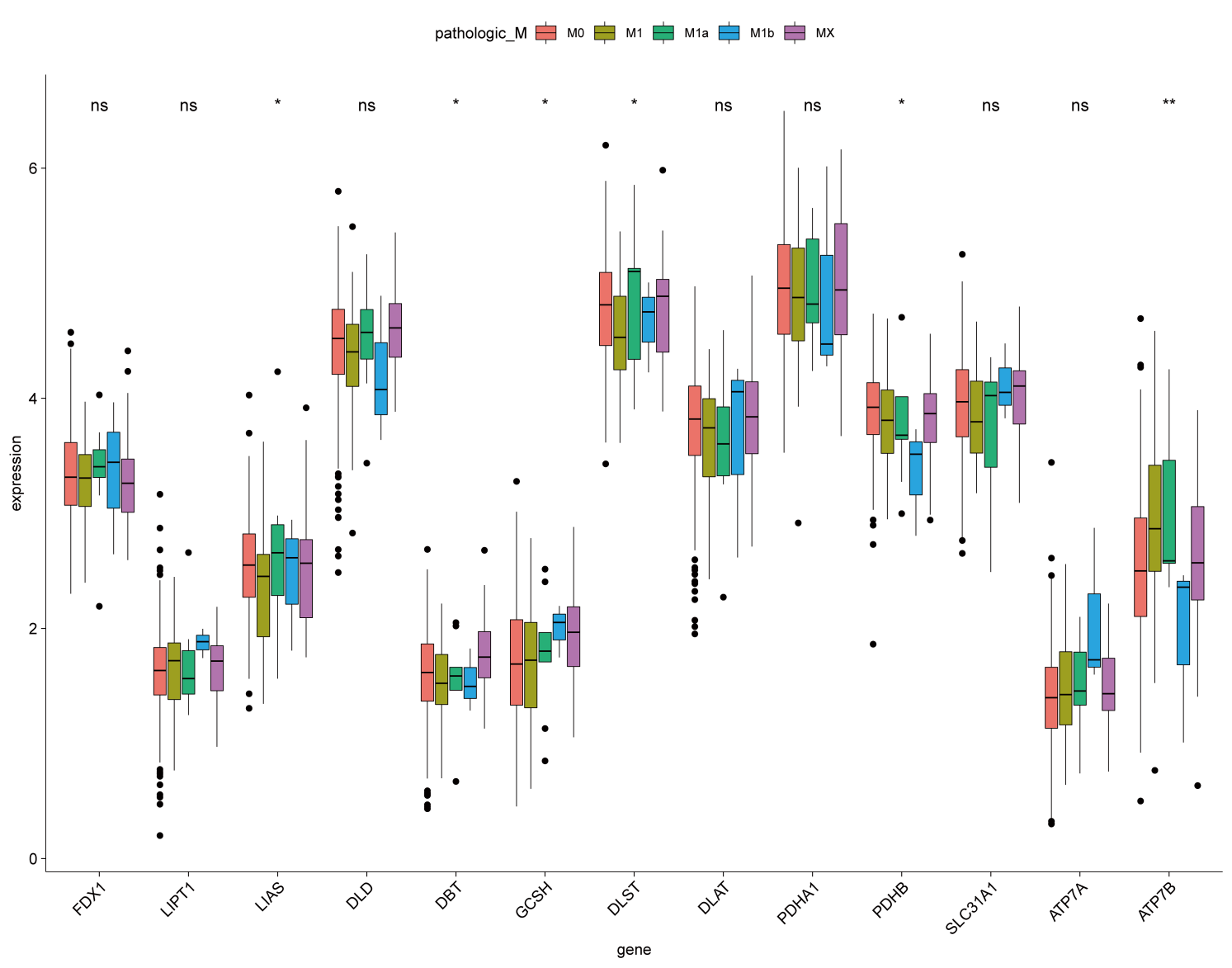
**

**Supplementary Figure S2.** The expression level of CRGs at different Node stages in COAD. ^ns^not significant.


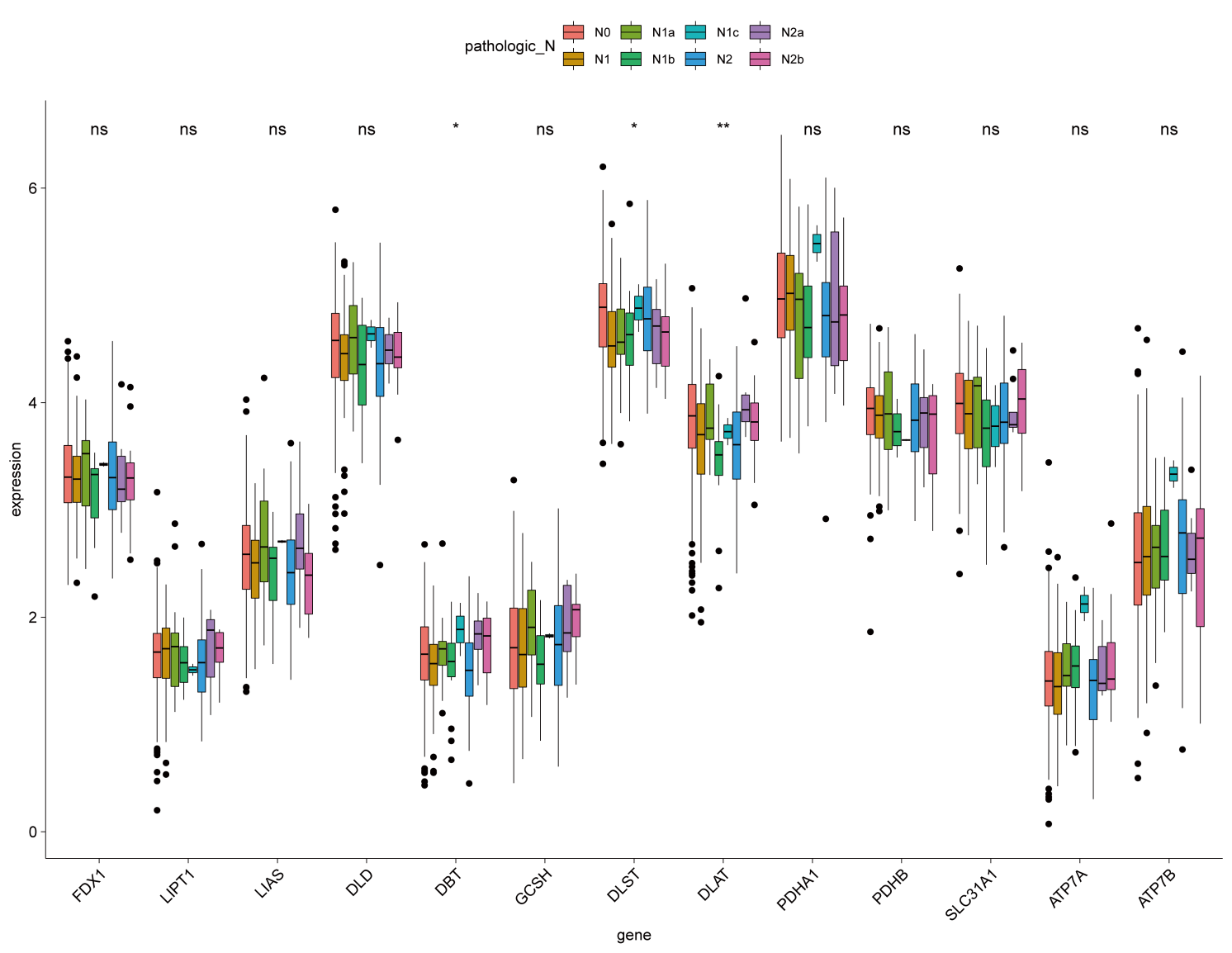


**Supplementary Figure S3.** The expression level of CRGs at different Metastasis stages in COAD. ^*^*p*<0.05, ^**^*p*<0.01, ^ns^not significant.

**
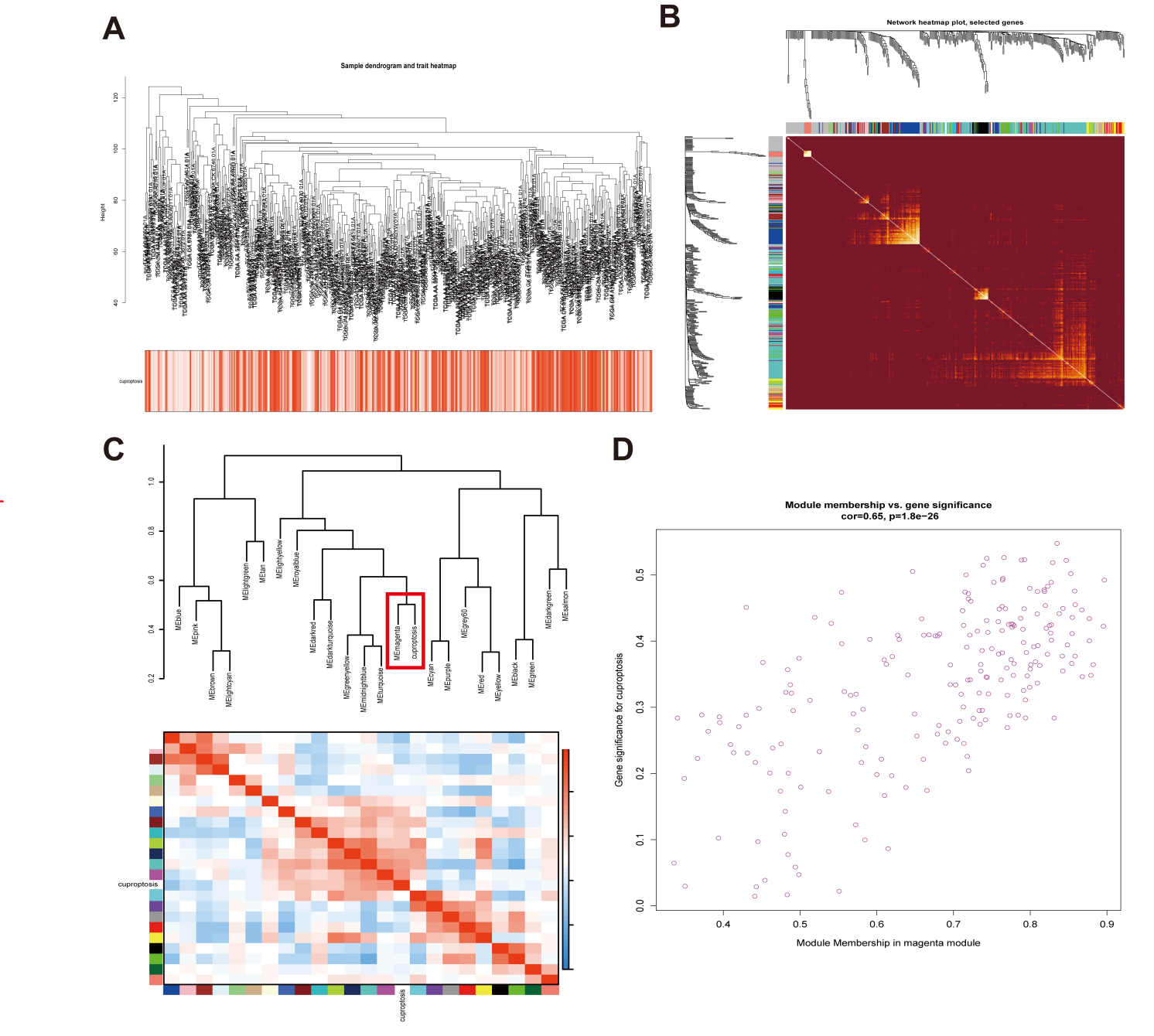
**

**Supplementary Figure S4. (A)** Sample dendrogram and trait heatmap demonstrated sample similarities and characteristics. **(B)** TOM-based gene correlation heatmap revealed module interactions and independence. **(C)** Cluster plot analysis explored correlations between modules and cuproptosis. **(D)** MM-GS-scatterplot of magenta module.


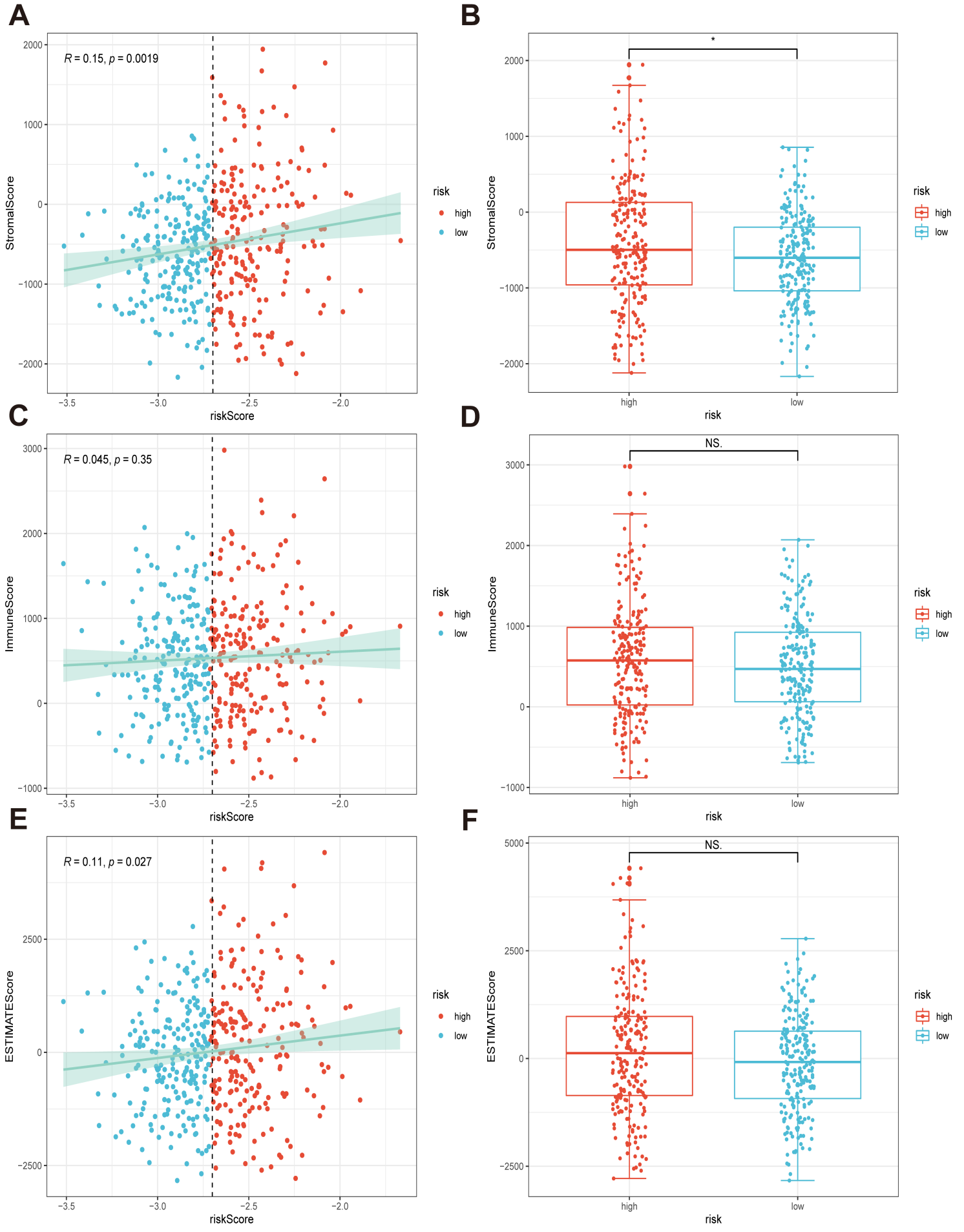


**Supplementary Figure S5. (A-F)** Relationships between expression of CRGs-related signature and ESTIMATE immune infiltration score.


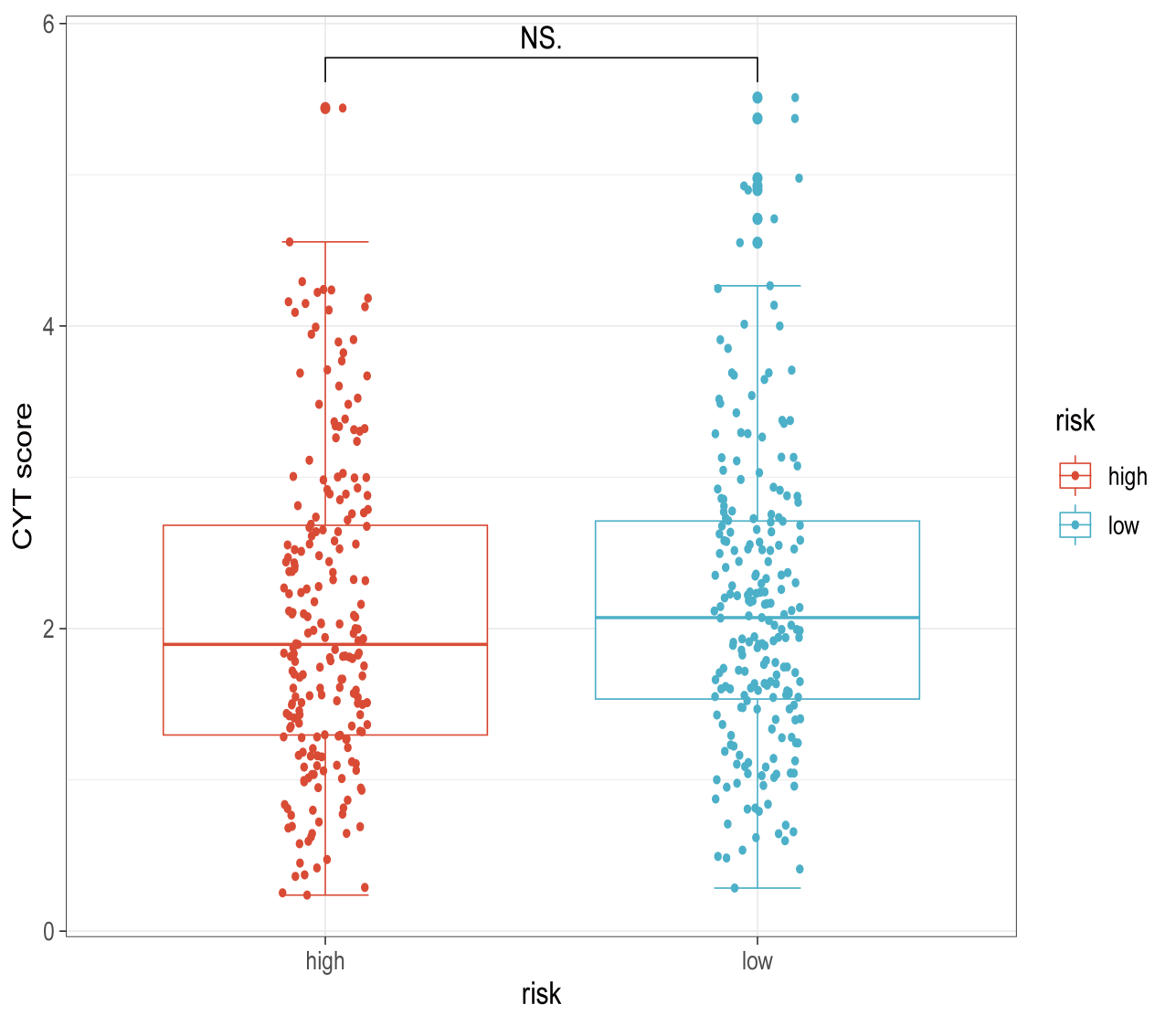


**Supplementary Figure S6.** Cytolytic activity score of high-risk and low-risk groups.


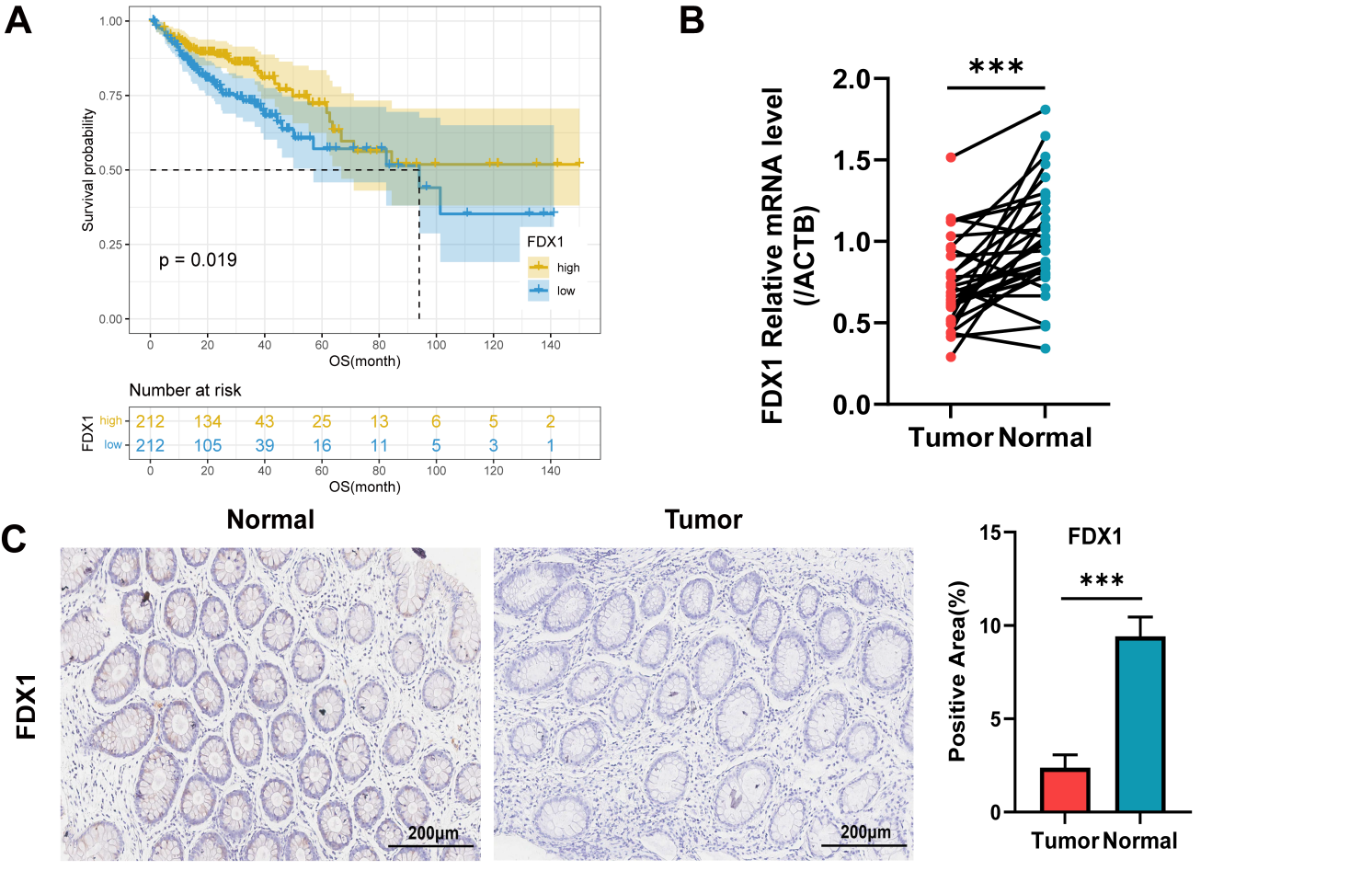


**Supplementary Figure S7.** FDX1 Expression in COAD and Its Impact on Prognosis. **(A)** Survival analysis of patients with high and low expression of FDX1. **(B)** Relative mRNA expression levels of FDX1 in cancer and paired para-cancer normal tissues from 30 COAD patients. **(C)** Immunohistochemical staining of FDX1 in COAD and normal colonic epithelial tissue. Scale bar is set at 200μm. ^***^*p*<0.001.


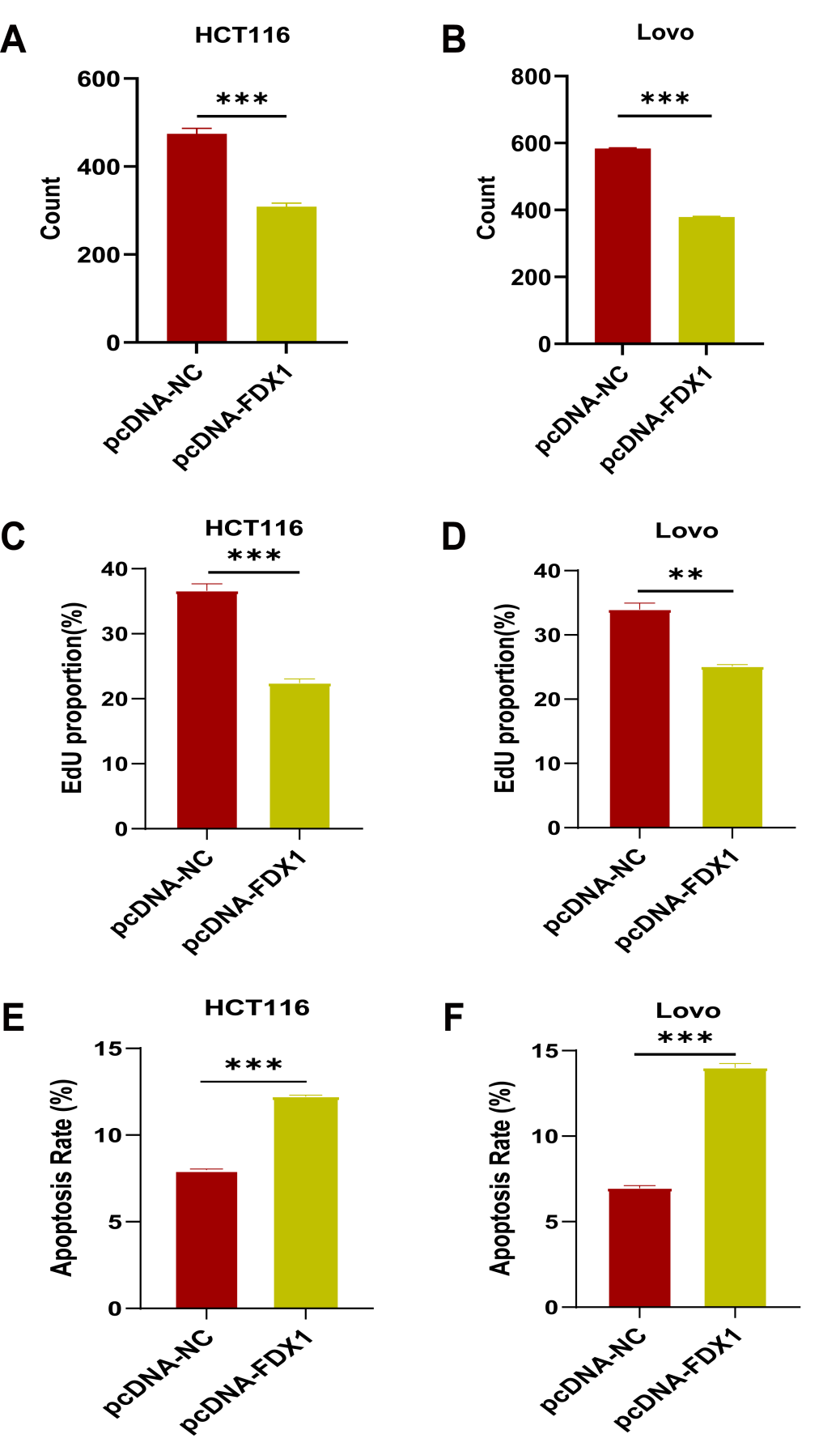


**Supplementary Figure S8. (A-B)** Counts of HCT116 and LoVo cell colonies after FDX1 overexpression in colony formation assay. **(C-D)** EdU proportion of HCT116 and LoVo cells after overexpressing FDX1. **(E-F)** The apoptosis rate of HCT116 and LoVo cells measured by flow cytometry after overexpressing FDX1. ^**^*p*<0.01, ^***^*p*<0.001.


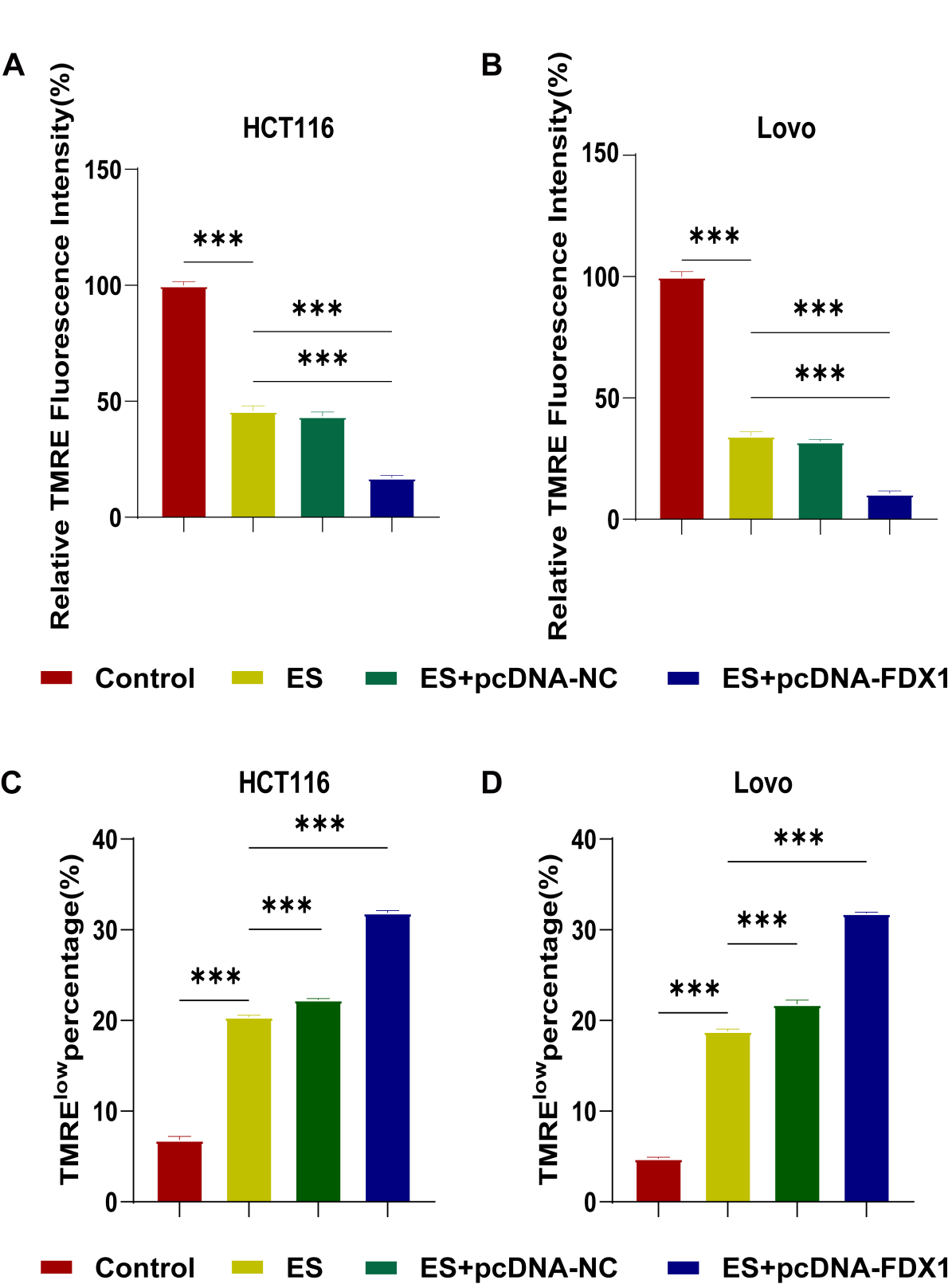


**Supplementary Figure S9. (A-B)** Statistical analysis of the fluorescence intensity of HCT116 and LoVo cells in Control, ES, ES+pcDNA-NC, and ES+pcDNA-FDX1 groups after TMRE staining. **(C-D)** Statistical analysis of flow cytometry data revealed the proportion of HCT116 and LoVo cells exhibiting decreased mitochondrial membrane potential after TMRE staining in the Control, ES, ES+pcDNA-NC, and ES+pcDNA-FDX1 groups. ^***^*p*<0.001.


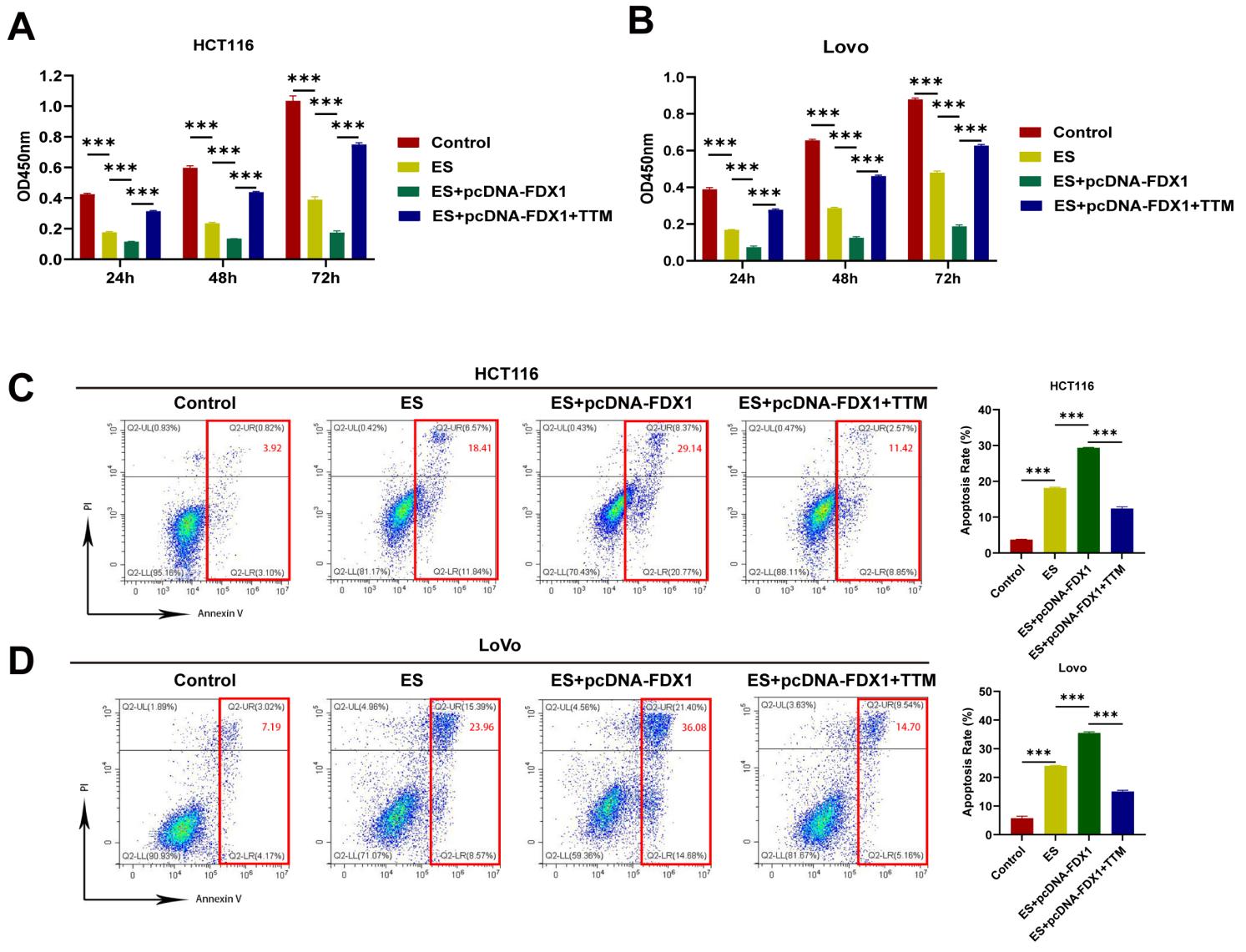


**SUPPLEMENTARY FIGURE S10. (A-B)** CCK-8 assay evaluated proliferation rates of HCT116 and LoVo cells in Control, ES, ES+pcDNA-FDX1, and ES+pcDNA-FDX1+TTM groups. **(C-D)** Flow cytometry analysis of apoptosis levels in HCT116 and LoVo cells from Control, ES, ES+pcDNA-NC, and ES+pcDNA-FDX1 groups. ^***^*p*<0.001.

## Supplementary Table

**Table S1 RT-qPCR amplification primers utilized in the study**

|  | FORWARD | REVERSE |
| --- | --- | --- |
| FDX1 | AACCCTGGCTTGTTCAACCTGTC | CCAACCGTGATCTGTCTGTTAGTCC |
| LIPT1 | TCGGAGAAGAAGTGGAGGAGGAAC | GGGGTTGGACAGCATTCAGAGC |
| DLD | GTCGTGTGTACTGCTCCTTGGC | AATCGGCTGATCTGCGTAAGTTCTC |
| DBT | GCACCACTGATCTTACAGGAGGAAC | GGCATTATCACTGGTTTGGCAAAGG |
| GCSH | CACTGGACCCGCTCTGCTCTC | AACAACATCTCCCAACGCTTCCTG |
| DLST | AGGGAGATGTCAGGTGGGAGAAAG | AAAGAGCTTCAATCACGCCATTTGC |
| DLAT | TTGATGTCAGTGTTGCGGTCAGTAC | GTGGCTGTAGTTTACCCTCTCTTGC |
| ATP7B | TCTGTGCTGATTGGAAACCGTGAG | CACCGTCAATAGCCACCAGGATG |
| ACTB | CATGTACGTTGCTATCCAGGC | CTCCTTAATGTCACGCACGAT |
